# Involvement of c-Myc/WWP1/TRIM65 Axis in Renal Fibrosis

**DOI:** 10.3390/biom16030373

**Published:** 2026-03-02

**Authors:** Sonia Mazumder, Cody Gifford, Jiaqi Tang, Fortis Gaba, Varsha Mondal, Roel Goldschmeding, Rohan Samarakoon, Paul J. Higgins

**Affiliations:** 1 Department of Regenerative and Cancer Cell Biology, Albany Medical College, 47 New Scotland Avenue, Albany, NY 12208-3479, USA; mazumds@amc.edu (S.M.); gifforc@amc.edu (C.G.); tangj@amc.edu (J.T.); mondalv@amc.edu (V.M.); 2 Department of Urology, Albany Medical College, 47 New Scotland Avenue, Albany, NY 12208-3479, USA; gabaf@amc.edu; 3 Department of Pathology, University Medical Center Utrecht, 3584 CX Utrecht, The Netherlands; rgoldsch@umcutrecht.nl

**Keywords:** SMAD1/5, SMAD3, BMP-7, PAI-1 CKD

## Abstract

Maladaptive tubular repair is a major contributor to fibrosis and chronic kidney disease (CKD), yet the molecular regulators of this process remain poorly understood. We report that the E3 ubiquitin ligases WWP1 and TRIM65 are novel regulators of tubular fibrosis. Both ligases were markedly induced in human and experimental CKD. WWP1 induction correlates with declining renal function in humans, highlighting the potential clinical relevance of WWP1. Profibrotic factor PAI-1 promotes a robust induction of WWP1 and TRIM65 in both primary human renal epithelial cells as well as cell line (HK-2). The silencing of WWP1 or TRIM65 significantly attenuated PAI-1-induced fibrotic signaling. Mechanistically, PAI-1 triggers a signaling cascade in which suppression of the regenerative BMP-7/SMAD5 pathway permits c-Myc induction, resulting in WWP1 and TRIM65 upregulation. The elevated expression of these ligases subsequently promotes epithelial dedifferentiation and fibrotic growth arrest. Restoration of BMP-7 or SMAD5 signaling disrupted this cascade and reduced fibrosis in renal tubular cells. Our study establishes a previously unrecognized PAI-1–c-Myc–WWP1/TRIM65 axis governing tubular maladaptive repair and positions WWP1 as a potentially new therapeutic target for slowing CKD progression.

## 1. Introduction

Chronic kidney disease (CKD) is now regarded as a hidden epidemic affecting around 40 million individuals in the US and 850 million people worldwide [1,2,3,4,5]. Irrespective of etiology, recurring kidney injury triggers tubular maladaptive repair (tubular dysfunction), which culminates in interstitial fibrosis, CKD, and end-stage renal disease (ESRD) [6,7]. Due to the lack of effective antifibrotic drug regimens, inadequate renal replacement availability, and the high prevalence of risk factors, CKD is projected to become the fifth leading cause of death by 2040, further adding to the increasing global medical and financial burden [1,2,3,4,5,8]. Tubular maladaptive repair in response to repetitive renal injury triggers epithelial dedifferentiation, G2/M arrest, induction of a senescence-like secretory phenotype (SASP), inflammatory cytokine secretion and pathogenic epithelial-fibroblast crosstalk leading to myofibroblast accumulation, interstitial fibrosis, and progressive nephron loss [9,10,11,12].

The involvement of the ubiquitin–proteasome system (UPS) during CKD progression is far less understood, although aberrant expression and/or activity of UPS components such as E3 ligases are linked to the progression of neoplastic and fibrotic diseases [13,14]. Consistent with the causative role of certain E3 ligases in CKD [13,14,15,16,17], the clinical-grade proteasome inhibitor Bortezomib protects mice from progressive aristolochic acid nephropathy (AAN) [18]. E3 ligases are involved in protein ubiquitination, although their functions are not limited to protein degradation [19]. The WW domain-containing E3 ubiquitin protein ligase 1 (WWP1) is highly upregulated in numerous cancers and drives tumor progression [20,21]. Although mice with WWP1 global ablation develop less cardiac hypertrophy and attenuated fibrotic ECM remodeling following pressure overload [22], WWP1 involvement in progressive nephropathies is not defined. The tripartite motif-containing (TRIM) family of E3 ubiquitin ligase proteins are involved in diverse cellular processes including cell cycle regulation and oncogenesis [23,24]. Among them, TRIM65 upregulation has been linked to carcinogenesis [25]. Although global silencing of TRIM65 in mice affords protection from renal fibrosis in general [26], its specific role as well as the mechanism of its induction during renal tubular injury is not well studied. A critical gap in the field is the identification of E3 ubiquitin ligases that integrate upstream fibrotic signals (such as transforming growth factor-β1/TGF-β1 and plasminogen activator inhibitor-1/PAI-1) and the loss of renal regeneration pathways (e.g., bone morphogenetic protein-7/BMP-7) that contribute to the establishment of a maladaptive renal epithelial state.

Among profibrotic networks, TGF-β1 is a well-known promoter of CKD via SMAD3 and non-SMAD3 transcription-factor-dependent mechanisms [15,27,28,29,30,31,32,33,34,35]. The expression of PAI-1, a major TGF-β1 target, is persistently upregulated in renal tubules regardless of renal insults [27,36]. Mice with PAI-1 ablation are, indeed, protected from renal fibrosis resulting from ureteral obstruction and diabetes, establishing PAI-1 as a CKD-promoting entity [37,38]. Although previous studies linked PAI-1 upregulation to extracellular matrix stabilization during fibrotic diseases, we were the first to implicate PAI-1 as a novel inducer of tubular maladaptive repair via p53 and pSMAD3 hyperactivation [39], two key transcription factors critical for renal maladaptive repair [35,40]. Activation of the transcription factor c-Myc in the kidney promotes fibrotic gene expression and CKD progression [41]. Whether c-Myc acts as a bridge between PAI-1 and downstream post-translational regulators such as E3 ligases, however, has not been addressed.

In contrast, bone morphogenetic protein-7 (BMP-7) and its downstream effectors SMAD1/5 comprise a well-established antifibrotic pathway in the kidney. BMP-7 promotes regenerative repair and counteracts TGF-β1–induced fibrosis by activating SMAD1/5 signaling [42,43,44,45]. During renal injury, loss of BMP-7/SMAD1/5 signaling shifts the balance toward fibrosis via hyperactivation of TGF-β1/SMAD3 pathway [46,47]. Whether BMP-7 suppression affects the activation of other profibrotic networks, including c-Myc and E3 ligases, remains unclear.

Collectively, these findings highlight a complex and poorly understood interplay among profibrotic (TGF-β1/PAI-1/c-Myc) and regenerative (BMP-7/SMAD1/5) networks that govern tubular cell fate and fibrosis progression during kidney injury. However, the mechanisms by which these opposing pathways converge on post-translational regulators such as E3 ubiquitin ligases remain unclear. To address this gap, we hypothesize that WWP1 and TRIM65 are induced by PAI-1- and c-Myc-dependent mechanisms during maladaptive tubular repair and that suppression of the BMP-7 signaling facilitates their activation. We investigated WWP1 and TRIM65 expression and regulation in experimental models of kidney fibrosis and human CKD transcriptomic datasets and examined their relationships to upstream fibrogenic and regenerative pathways to define novel mechanisms driving CKD progression.

## 2. Materials and Methods

### 2.1. Sex as a Biological Variable and Exclusion/Inclusion Criteria

All animal studies included both genders. All human samples from the Nephroseq (https://www.nephroseq.org (accessed on 5 June 2024)) database and single cell renal atlas (Accession No.: GSE183279) [48] include both males and females. In this study, sex was not considered as a biological variable. No exclusion and inclusion criteria were used for animal or cell culture studies.

### 2.2. Animals

C57Bl/6 mice were purchased from Charles River Laboratories (https://www.criver.com/ (Wilmington, MA, USA)). The mice were maintained in pathogen-free SPF conditions with a 12 h light–dark cycle and a constant environmental temperature. Mice were fed a standard pellet diet with free access to water. The sample sizes for our animal experiments were chosen based on our prior experience and previously published studies [30,49,50,51].

### 2.3. Unilateral Ureteral Obstruction (UUO) Nephropathy

Briefly, C57Bl/6 mice (6–8-weeks-old with a similar weight range) were anesthetized using isoflurane inhalation and under aseptic conditions, a small incision was made in the flank region to expose the kidneys. The ureter of the left kidney was ligated with two 5-0 silk sutures to generate a renal obstruction, and the unmanipulated contralateral right kidney served as a control. All animals were euthanized on day 7 post-ligation, and obstructed (UUO) and contralateral kidneys were harvested for biochemical analysis. The approximate mortality rate for the UUO procedure is 20%. The experiments were carried out in accordance with the European Community Guidelines and in compliance with the protocols approved (Approval no.: 2009.II.11.129) by the Animal Experiments Committee (DEC) of the University of Utrecht (Utrecht, The Netherlands).

### 2.4. Aristolochic Acid Nephropathy (AAN)

C57Bl/6 mice (6–8-weeks-old with a similar weight range) were intraperitoneally injected with aristolochic acid (AA) sodium salt (5 mg/kg body weight dissolved in distilled water; A9451; Sigma-Aldrich, St. Louis, MO, USA) once a day for 5 consecutive days (AAN group), while control mice received a NaCl vehicle (NaCl group). At 25 days, following the initial injections, all mice were euthanized, and the kidneys were harvested for biochemical analysis. The mortality rate for mice subjected to AAN is around 10%. All animals were randomly assigned to experimental (AAN group) and control groups (NaCl group). The experiments were conducted in compliance with the ethical guidelines and protocols approved (Approval no.: 2011.II.05.086) by the Animal Experiments Committee (DEC) of the University of Utrecht (Utrecht, The Netherlands).

### 2.5. Human Kidney Specimens

Diabetic renal tissue was obtained from a donor deemed unsuitable for transplantation due to advanced diabetic nephropathy, characterized by histological features such as Kimmelstiel–Wilson nodules. Normal human kidney tissue was collected from the non-neoplastic region of a nephrectomy specimen from a patient undergoing surgery for renal cell carcinoma. All patient samples were leftover body material from clinical biopsies (or resections) and were collected according to the ethical guidelines of the University of Utrecht (Utrecht, The Netherlands). Samples were anonymized, allowing us to use this residual tissue for research purposes without the consent of the patients.

### 2.6. Cell Culture and Generation of Stable Single and Double Transductants

Human renal proximal tubular epithelial cells (HK-2) (CRL-2190- ATCC, Manassas, VA, USA) were grown in DMEM media M (1X) + GlutaMAX-I (10567-014, Gibco, Thermo Fisher Scientific, Waltham, MA, USA), 5% FBS (fetal bovine serum) (16000-044, Gibco, Thermo Fisher Scientific, Waltham, MA, USA), and 5 units/mL penicillin + 5 µg/mL streptomycin (15140-122, Gibco, Thermo Fisher Scientific, Waltham, MA, USA). Primary human tubular epithelial cells (RPTECs, #CC-2553) were purchased from Lonza Biosciences (Walkersville, MD, USA). In order to generate PAI-1 stable transductants, semiconfluent HK-2 cultures were infected with lentiviruses carrying a cytomegalovirus (CMV) promoter–driven PAI-1 cDNA construct (termed CMV-PAI-1) (LPP-F0606-Lv105) or an empty vector (termed CMV-Con) (LPP-NEG-Lv105) (GeneCopoeia, Rockville, MD, USA) in the presence of Polybrene at 5 μg/mL (sc-134220, Santa Cruz Biotechnology, Dallas, TX, USA) in 5% FBS/DMEM for 24 h. After a 24 h recovery, the cells were subjected to stable selection in complete medium (1X DMEM + GlutaMAX-I; 5% FBS; 5 units/mL penicillin + 5 µg/mL streptomycin) containing 5 µg/mL puromycin dihydrochloride (sc-108071, Santa Cruz Biotechnology, Dallas, TX, USA). Media were changed every 3 days to maintain the appropriate selection pressure. To generate RPTEC transgenic cells overexpressing PAI-1, cells were transiently infected with the LPP-F0606-Lv105 or control LPP-NEG-Lv105 lentiviral constructs (GeneCopoeia, Rockville, MD, USA) for 5 days prior to lysate collection. PAI-1 induction in transgenic cell types was confirmed by immunoblot analysis. Due to the proliferative defects following stable PAI-1 expression in HK-2 epithelial cells, CMV-PAI-1 cultures were initially seeded at a density three times higher than CMV-Con plates to achieve equivalent cell numbers at the time of lysate generation for Western blot analysis.

To generate stable double-transgenic epithelial cell lines for BMP-7 and SMAD5 expression rescue experiments, semiconfluent PAI-1 stable transductants were reinfected with CMV-BMP-7-GFP (LPP-A0309-Lv122) and CMV-SMAD5-GFP (LPP-I0510-Lv122) lentiviral particles, respectively, or CMV-Control vector lentiviral particles (LPP-NEG-Lv105) (GeneCopoeia, Rockville, MD, USA) for 1–2 days in complete medium with Polybrene at 5 μg/mL before stable selection. Successful restoration of BMP-7 and SMAD5 expression rescue was confirmed by immunoblotting for the GFP. To create stable double-transgenic epithelial cell lines with WWP1, c-Myc, and TRIM65 silencing in the context of PAI-1 upregulation, 40% confluent CMV-PAI-1 cultures were reinfected with either control (sc-108080) or WWP1 (sc-40366-V), c-Myc (sc-29226-V), and TRIM65 (sc-93883-V) short hairpin RNA (shRNA) lentiviral constructs (Santa Cruz Biotechnology, Dallas, TX, USA) for 1–2 days in complete medium containing Polybrene at 5 μg/mL followed by stable selection. WWP1, c-Myc, and TRIM65 depletion in PAI-1-overexpressing double-transgenic cultures were confirmed by Western blot analysis for respective proteins. WWP1-GFP expression lentiviral particles were purchased from Origene (RC206243L4, Rockville, MD, USA). All cells were routinely checked for contamination. Where appropriate, the blinding of the investigators was used as a strategy to reduce experimental bias. All cell culture experiments were repeated three times.

### 2.7. Western Blot Analysis and Antibodies

Cells were lysed in Laemmli sample buffer containing 5% β-mercaptoethanol and boiled for 5 min. Kidney tissues were extracted in 2% SDS/PBS. For SDS-PAGE electrophoresis, approximately 20–30 μg of protein from each sample was loaded into Bio-Rad Mini-PROTEAN TGX 10% pre-cast gels (4561034; Bio-Rad Laboratories, Hercules, CA, USA). Separated proteins were transferred to nitrocellulose membranes (1620112; Bio-Rad Laboratories, Hercules, CA, USA) and blocked in 5% non-fat dry milk in 0.05% Triton-X 100/PBS buffer. The membranes were then probed with the following primary antibodies overnight: rabbit anti-fibronectin (1:50,000; ab2413), rabbit anti-tubulin (1:20,000; ab6046), rabbit anti-phospho-SMAD3 (1:1000; ab52903), rabbit anti-WWP1 (1:1000; ab43791), rabbit anti-c-Myc (1:2000; ab32072), rabbit anti-BMP-7 (1:1000; ab84684), rabbit anti-osteopontin (1:2000; ab8448) from Abcam (Cambridge, UK), rabbit anti-vimentin (1:10,000; sc5565), mouse anti-p53 (1:500; sc126), mouse anti-GFP (1:500; sc-9996), goat anti-CTGF (1:1000; sc14939) from Santa Cruz Biotechnology (Dallas, TX, USA), rabbit anti-p21 (1:1000; 2947), rabbit anti-SMAD5 (1:1000; 12534), rabbit anti-phospho-SMAD1/5 (1:1000; 9516), rabbit anti-snail (1:1000; 3879), rabbit anti-phospho Histone H3 (1:1000; 9701) from Cell Signaling Technology (Danvers, MA, USA), mouse anti-E-cadherin (1:1000; 610181) from BD Biosciences, mouse anti-TRIM65 (1:250; H00201292-B01P) from Novus Biologicals, rabbit anti-collagen type 1 (1:5000; 234167) from Calbiochem, and rabbit anti-PAI-1 (1:3000) as described previously [39]. Membranes were washed three times and incubated with appropriate HRP-conjugated secondary antibodies (goat anti-rabbit, 31460; goat anti-mouse, 31430), both from Thermo Fisher Scientific (Waltham, MA, USA), and mouse anti-goat, sc-2354 from Santa Cruz Biotechnology (Dallas, TX, USA) at a dilution of 1:1000–5000 for 1 h at room temperature. Following three consecutive washes in 0.05% Triton-X 100/PBS, membranes were incubated in ECL (Bio-Rad Clarity Western ECL Substrate; 170-5061; Bio-Rad Laboratories, Hercules, CA, USA) and imaged with a ChemiDoc^TM^ Imaging system (Bio-Rad Laboratories, Hercules, CA, USA). Relative protein levels were quantified using the ImageJ software package (version 1.53t) (National Institute of Health, MD, USA). All the antibodies are commercially available and validated by the manufacturers and/or us.

### 2.8. Immunofluorescence

CMV-Con and CMV-PAI-1 cells were plated on coverslips in a 6-well culture chamber and allowed to reach 70–80% confluency. Cells were then washed 2 times with 1X PBST (1X PBS; Dulbecco’s phosphate buffer saline, 14190-144, Gibco, Thermo Fisher Scientific, Waltham, MA, USA + 0.05% tween-20; P7949, Sigma-Aldrich, MO, USA) followed by fixation with 4% paraformaldehyde (J61899.AP, Thermo Fisher Scientific, Waltham MA, USA) for 5 min and permeabilized with 0.5% Triton X-100 (T9284, Sigma-Aldrich, MO, USA) in 1X PBST for 10 min at room temperature. The cells were then blocked in 1% BSA in 1X PBST for 60 min in a humidity chamber (incubator) (Eppendorf, Hamburg, Germany) with occasional agitation followed by incubation with primary antibodies to rabbit anti-WWP1 (1:250; Abcam-ab43791) and rabbit anti-c-Myc (1:250; Abcam-ab32072) overnight at 4 °C. The cells were then washed three times (5 min each) before incubating with Alexafluor 647 (1:1000; A-21245; Invitrogen, CA, USA) and Alexafluor 594 (1:1000; A-11037; Invitrogen, CA, USA) secondary antibody, respectively, for 1 h in a humidity chamber in the dark. After washing, Hoechst stain in 1X PBST (1:15,000; H-3569, Molecular probes, OR, USA) was added to the cells for nuclear staining and incubated for 5 min with agitation at room temperature before coverslips were mounted using Prolong^TM^ Diamond anti-fade mounting media (P36961; Invitrogen, CA, USA). After 24 h post-curation, images were acquired at 40× magnification using a Nikon Eclipse Ti2-E inverted microscope operated by NIS elements software (version 5.41.2.17110) (Tokyo, Japan).

### 2.9. Analysis of Single Cell RNA-Seq Dataset on Human Renal Heathy and Diseased Specimens

One human renal atlas (Accession No.: GSE183279) [48], including specimens from several renal disease and healthy patients’ kidneys, was used in our study, and the original cell annotation provided by authors was used for downstream analysis. Dataset GSE183279 is one of the biggest renal cellular atlases, including 58 reference tissues and 52 diseased tissues. The raw count matrix and annotated metadata for single cell RNA sequencing dataset (Accession No.: GSE183279) were downloaded from the Gene Expression Omnibus (GEO) (https://www.ncbi.nlm.nih.gov/geo/ (accessed on 4 February 2025), followed by the creation of a Seurat object by merging both the count matrix and annotated data together. A series of downstream quality control steps were performed as listed by the authors [48], including filtering out low-quality cells or potential doublets (a cut-off of <50% mitochondrial reads per cell; >500 and <5000 genes per cell were applied), normalization, scaling, and identifying highly variable genes. All highly variable genes were utilized for linear dimensionality reduction (PCA, principal component analysis). Uniform manifold approximation and projection (UMAP) non-linear dimensionality reduction was performed using the top 50 principal components identified. Followed by dimensionality reductions, UMAP plots for annotated cell clusters and target gene expression were generated. All data processing, analysis, and visualization steps were performed using the R programming language (R software: version R 4.4.3) in the R studio environment (version 2024.12.1+563). Key packages utilized in this process included Seurat (version 5.2.1), ggplot2 (version 3.5.1), and dplyr (version 1.1.4). This study included all specimens described in the dataset (Accession No.: GSE183279) by the authors [48].

### 2.10. Analysis of Renal Disease Datasets from Nephroseq

Renal transcriptomics from the Nephroseq database (https://www.nephroseq.org (accessed on 5 June 2024)) were utilized for analyzing the mRNA expression of genes of interest. A minimum fold change of 1.5 and *p*-value of <0.05 were applied. The log2 median-centered intensity or expression estimates for both the healthy control and disease groups for the respective datasets (ERCB Nephrotic Syndrome TubInt, ERCB Lupus TubInt, Nakagawa CKD Kidney [52], Woroniecka Diabetes TubInt [53], and Ju CKD TubInt [54]) were downloaded from the Nephroseq database (https://www.nephroseq.org (accessed on 5 June 2024)). The expression estimates were then utilized to generate the histograms using GraphPad Prism (GraphPad Software, Inc., Boston, MA, USA) (version 10). The graphs and correlation analysis between WWP1 log2 expression values and GFR or proteinuria or serum creatinine levels were performed using the R programming language (version R 4.4.3) in the R studio environment (version 2024.12.1+563).

### 2.11. Statistical Analysis

Statistical differences between the biological groups were assessed using two-tailed Student’s *T*-tests or a one-way ANOVA followed by a Tukey’s post-hoc test, as appropriate. A *p*-value of less than 0.05 was considered statistically significant. All histograms and statistical plots were generated using GraphPad Prism (version 10; GraphPad Software, Inc., Boston, MA, USA). A Pearson correlation analysis was used to assess the relationship between WWP1 expression and GFR, serum creatinine levels, and proteinuria in human CKD patients using the R programming language (R software: version R 4.4.3) in the R studio environment (version 2024.12.1+563).

## 3. Results

### 3.1. WWP1 and TRIM65 E3 Ligases Are Highly Upregulated in Humans During Renal Injury, and Renal Upregulation of WWP1 Positively Correlates with Chronic Kidney Disease

**Figure 1:**
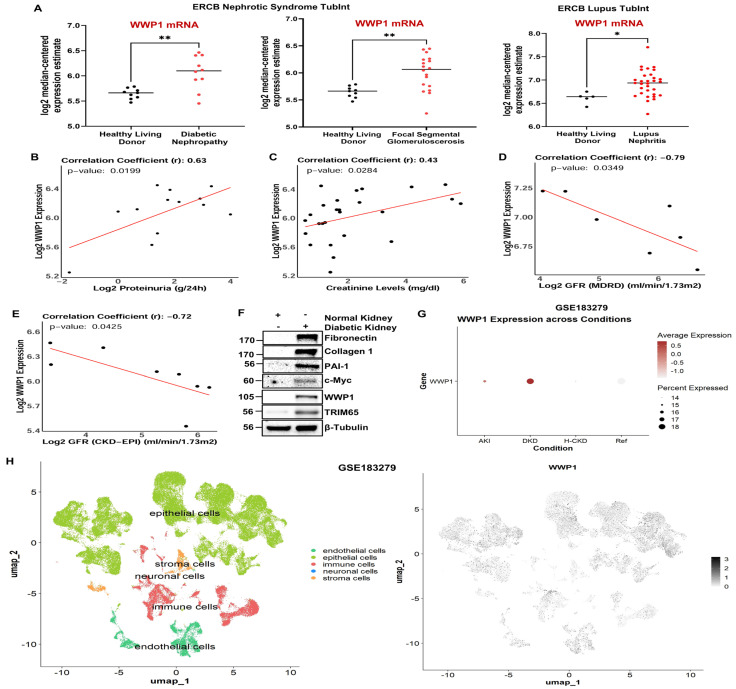
WWP1 is highly upregulated in fibrotic human kidneys and correlates with a decline in renal function and disease progression. Renal disease datasets (ERCB Nephrotic Syndrome TubInt, ERCB Lupus TubInt) available from Nephroseq (https://www.nephroseq.org (accessed on 5 June 2024)) (**A**) were analyzed for WWP1 mRNA levels in human diabetic, FSGS (focal segmental glomerulosclerosis), and lupus nephritis kidneys relative to healthy controls. Data in (**A**) are represented as the median. * *p* < 0.05, ** *p* < 0.01. Human renal disease specimens from (**A**) were further assessed for a correlation analysis between WWP1 expression and proteinuria (**B**) (r = 0.63, *p* = 0.0199) (ERCB Nephrotic Syndrome TubInt), or serum creatinine level (**C**) (r = 0.43, *p* = 0.0284) (ERCB Nephrotic Syndrome TubInt), or glomerular filtration rate (GFR) (**D**,**E**) (r = -0.79, *p* = 0.0349; r = -0.72, *p* = 0.0425) (ERCB Nephrotic Syndrome TubInt, ERCB Lupus TubInt). Lysates from human healthy and diabetic kidneys were immunoblotted for fibronectin, collagen 1, PAI-1, c-Myc, WWP1, and TRIM65 (**F**) proteins. A single cell RNA sequencing dataset (Accession No.: GSE183279) was analyzed for WWP1 transcript levels in the diseased kidneys relative to the reference (healthy controls) (**G**) represented as a dot plot, where the intensity of dot color (brown) dictates the WWP1 expression level, and dot size represents the percentage of cells expressing WWP1. UMAP analysis of the dataset (Accession No.: GSE183279) was performed to determine the compartment-specific expression of WWP1 in the kidney (**H**), where in the left panel, yellow-green represents epithelial cells, sea green represents endothelial cells, red represents immune cells, orange represents stroma cells, and a very small percentage of neuronal cells are represented by the blue color.

Although recent studies suggest that proteosome inhibitors attenuate progression of certain chronic kidney diseases in mice [18], the identity of the ubiquitin ligases involved are not well understood. Therefore, we sought to identify novel E3 ligases important in renal tubular maladaptive repair and fibrosis and to characterize their regulation and associated pathogenic mechanisms. To investigate the role of WWP1 in renal fibrosis progression, we first analyzed its expression profile in healthy vs. renal disease specimens. An analysis of the available bulk RNA sequencing and human renal disease transcriptome datasets from Nephroseq (https://www.nephroseq.org (accessed on 5 June 2024)) revealed that WWP1 (WW domain-containing E3 ubiquitin protein ligase 1) mRNA expression is significantly upregulated in several progressive kidney disease types, including diabetic nephropathy (DN), focal segmental glomerulosclerosis (FSGS), and lupus nephritis (Figure 1A), compared to respective healthy human kidneys. A reduction in glomerular filtration rate (GFR) and elevated serum creatinine levels, as well as proteinuria (presence of protein in the urine), are widely used indicators of CKD progression. Our analysis revealed that WWP1 expression positively correlates with proteinuria (Figure 1B) and serum creatinine levels (Figure 1C), suggesting that WWP1 could contribute to the progression of renal injury. Moreover, WWP1 mRNA levels inversely correlate with glomerular filtration rate (GFR) (Figure 1D,E), indicating that WWP1 induction strongly associates with the decline in renal function. Immunoblot analysis of human diabetic kidneys, indeed, confirmed robust WWP1 protein induction in the fibrotic kidneys compared to respective controls (Figure 1F). Further analysis of a recent single cell RNA sequencing dataset (Accession No.: GSE183279) [48] from human renal specimens revealed that WWP1 mRNA expression is induced in the renal epithelial compartments of the diabetic kidneys (Figure 1G,H), which suggests potential involvement of WWP1 in tubular pathologies. Transcript levels of TRIM65 are also highly induced in human CKD patients compared to control groups as determined by analyzing the similar annotated single cell RNA sequencing human renal disease dataset (Accession No.: GSE183279) [48].

Western blot analysis of human diabetic kidneys further confirmed a dramatic increase in TRIM65 protein levels in fibrotic kidneys compared to healthy controls (Figure 1F). Although a recent study indicates that TRIM65 attenuates fibrotic lesions in obstructive and folic acid nephropathies in mice [26], whether this ligase impacts tubular pathologies during CKD progression is not defined.

### 3.2. WWP1 and TRIM65 Are Dramatically Upregulated in Mouse Fibrotic Kidneys Undergoing Ureteral Unilateral Obstruction (UUO) and Aristolochic Acid Nephropathy (AAN)

**Figure 2:**
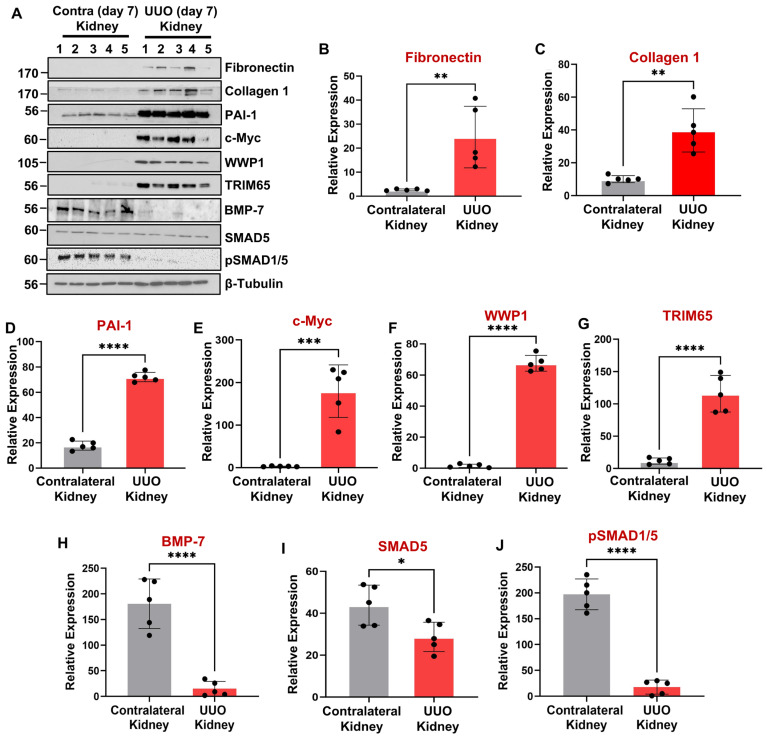
PAI-1, WWP1, TRIM65, and c-Myc upregulation correlated with repression of the BMP-7/SMAD1/5 signaling axis in fibrotic UUO kidneys. Mice were subjected to unilateral ureteral obstruction (7 days) prior to the extraction of obstructed (UUO) and contralateral kidneys. Contralateral and UUO renal extracts were assessed for fibronectin (**A**,**B**), collagen 1 (**A**,**C**), PAI-1 (**A**,**D**) and c-Myc (**A**,**E**), WWP1 (**A**,**F**), and TRIM65 (**A**,**G**), BMP-7 (**A**,**H**), SMAD5 (**A**,**I**), and pSMAD1/5 (**A**,**J**) protein levels by immunoblot analysis. β-tubulin is serving as a loading control, and the expression of each indicated protein is normalized to tubulin level. Histograms (**B**–**J**) depict the expression (mean ± SD) comparisons for the indicated protein in the UUO and contralateral kidneys (reference) using a Student’s *T*-test for 5 animals per group shown as 1–5 in (**A**). * *p* < 0.05, ** *p* < 0.01, *** *p* < 0.001, **** *p* < 0.0001.

**Figure 3:**
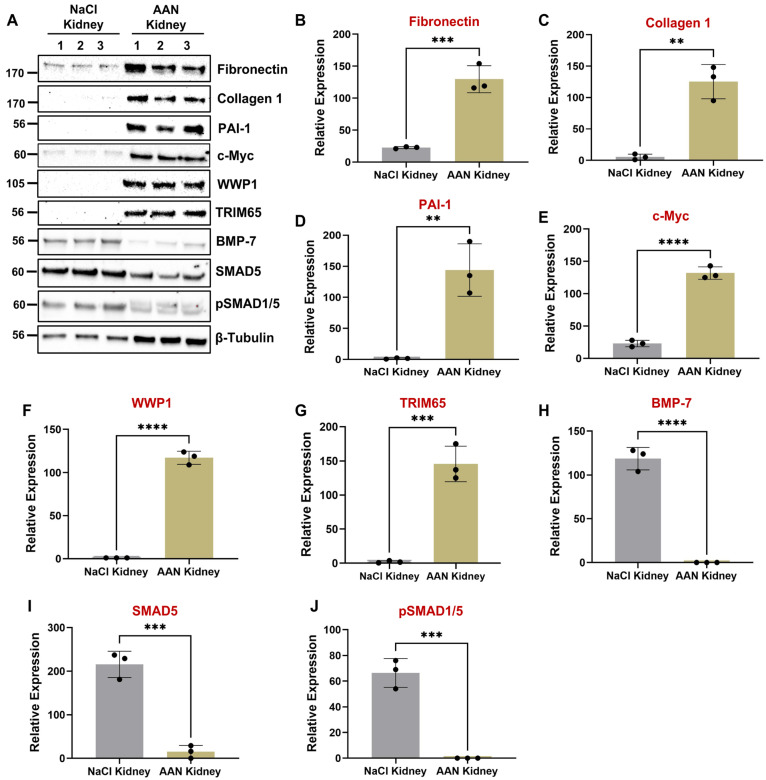
Induction of PAI-1, WWP1, TRIM65, and c-Myc correlates with BMP-7/SMAD1/5 signaling axis deregulation in aristolochic acid (AA) induced fibrotic kidneys. Mice were administered either with a NaCl vehicle (control) or aristolochic acid (AA) sodium salt (5 mg/kg body weight dissolved in distilled water) via intraperitoneal injection, daily for 5 consecutive days, and termed as NaCl kidney and AAN kidney, respectively. Twenty-five days post-AA injections, mice in both groups were euthanized for kidney harvesting. Renal lysates from both groups were western blotted for fibronectin (**A**,**B**), collagen 1 (**A**,**C**), PAI-1 (**A**,**D**), c-Myc (**A**,**E**), WWP1 (**A**,**F**), TRIM65 (**A**,**G**), BMP-7 (**A**,**H**), SMAD5 (**A**,**I**), and pSMAD1/5 (**A**,**J**). Data are presented as the mean ± SD. The expression of each indicated protein is normalized to β-tubulin (loading control). Histograms (**B**–**J**) depict the expression comparisons for the indicated proteins in the AAN and NaCl (reference) kidneys using a Student’s *T*-test for 3 animals per group shown as 1–3 in (**A**). ** *p* < 0.01, *** *p* < 0.001, **** *p* < 0.0001.

UUO and AAN are established models for investigating tubular-injury-initiated progression to interstitial fibrosis [55,56,57] as confirmed by an increased expression of fibronectin (Figure 2A,B; Figure 3A,B), collagen 1 (Figure 2A,C; Figure 3A,C), and PAI-1 (Figure 2A,D; Figure 3A,D). Immunoblot analysis of renal extracts confirmed that WWP1 (Figure 2A,F; Figure 3A,F) and TRIM65 (Figure 2A,G; Figure 3A,G) are robustly upregulated in the UUO (7 days post-ligation) and AAN (25 days post-AA administration) kidneys relative to contralateral or vehicle-treated controls. Furthermore, PAI-1 upregulation in fibrotic human (Figure 1F) and mouse kidneys (Figure 2A,D; Figure 3A,D) correlates with WWP1 induction (Figure 1F; Figure 2A,F; Figure 3A,F), suggesting a possible causal relationship between these entities.

### 3.3. PAI-1 Promotes WWP1 and TRIM65 Upregulation to Trigger Tubular Dysfunction

**Figure 4:**
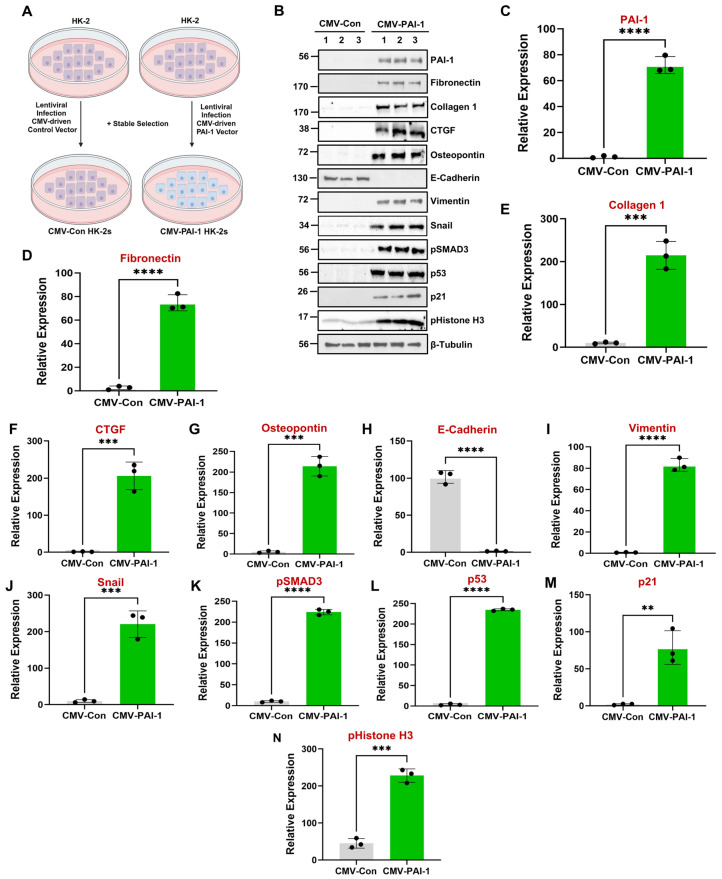
Sustained epithelial PAI-1 expression promotes maladaptive repair (tubular dysfunction). Schematic for the generation of PAI-1-overexpressing cells (**A**). Lysates of CMV-Con and CMV-PAI-1 cells were immunoblotted for PAI-1 (**B**,**C**), fibronectin (**B**,**D**), collagen 1 (**B**,**E**), CTGF (**B**,**F**), osteopontin (**B**,**G**), E-cadherin (**B**,**H**), vimentin (**B**,**I**), snail (**B**,**J**), pSMAD3 (**B**,**K**), p53 (**B**,**L**), p21 (**B**,**M**), pHistone H3 (**B**,**N**), and β-tubulin (loading control) (**B**). Histograms (**C**–**N**) depict the expression (mean ± SD) differences of PAI-1 (**C**), fibronectin (**D**), collagen 1 (**E**), CTGF (**F**), osteopontin (**G**), E-cadherin (**H**), vimentin (**I**), snail (**J**), pSMAD3 (**K**), p53 (**L**), p21 (**M**), and pHistone H3 (**N**) in the CMV-Con (reference) and CMV-PAI-1 cell populations in three independent experiments (*n* = 3) in triplicate. A Student’s *T*-test was used for statistical comparisons. ** *p* < 0.01, *** *p* < 0.001, **** *p* < 0.0001.

Our previous studies demonstrated that PAI-1 promotes tubular dysfunction via dedifferentiation, G2/M arrest, and the induction/secretion of ECM proteins, and the expression of profibrotic cytokines and growth factors, which is orchestrated by p53- and pSMAD3-dependent mechanisms [39]. Indeed, sustained PAI-1 upregulation (Figure 4B,C) results in a dramatic loss of E-cadherin (Figure 4B,H) and a significant upregulation of vimentin (Figure 4B,I) and snail (Figure 4B,J), indicative of dedifferentiation. Markers of cell cycle arrest p21 (Figure 4B,M) and pHistone H3 (Figure 4B,N) expression are also robustly induced following persistent PAI-1 induction. In addition, several fibrotic factors (Figure 4B, D-G) as well as pSMAD3 (Figure 4B,K) and p53 (Figure 4B,L) are significantly upregulated in the CMV-PAI-1 population relative to CMV-Con cultures. Notably, PAI-1 overexpression in primary human tubular epithelial cells (RPTECs) largely recapitulated the fibrotic tubular dysfunctional phenotype (Supplementary Figure S1).

**Figure 5:**
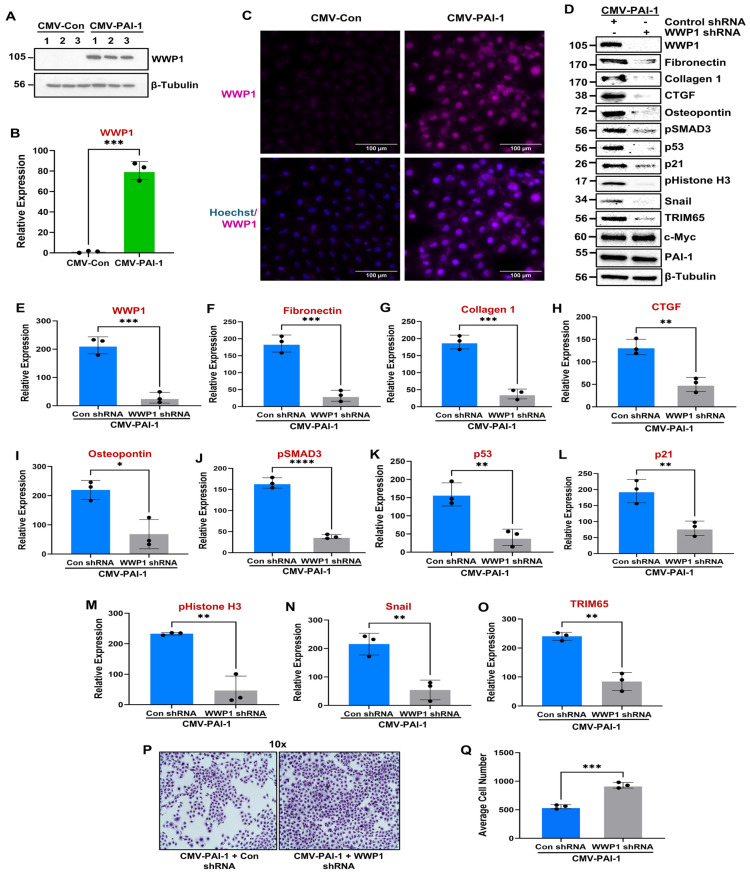
PAI-1-mediated WWP1 upregulation is causatively linked to tubular dysfunction. Western blot analysis of CMV-Con (reference) and CMV-PAI-1 cell lysates for WWP1 expression (**A**); the histogram in (**B**) represents the expression (mean ± SD) differences of WWP1 between groups for three independent studies (*n* = 3) in triplicate. *** *p* < 0.001. WWP1 expressions in CMV-Con and CMV-PAI-1 transgenic cell monolayers are confirmed in (**C**) immunofluorescence imaging, showing WWP1 (in magenta) and nuclear staining with Hoechst (in blue) (40× magnification and scale bars = 100 μm in (**C**), *n* = 3). CMV-PAI-1 cultures were stably infected with either control shRNA (reference) or WWP1 shRNA lentiviral particles and double transgenic lysates were subjected to western blot assessment for WWP1 (**D**,**E**), fibronectin (**D**,**F**), collagen 1 (**D**,**G**), CTGF (**D**,**H**), osteopontin (**D**,**I**), pSMAD3 (**D**,**J**), p53 (**D**,**K**), p21 (**D**,**L**), pHistone H3 (**D**,**M**), snail (**D**,**N**), TRIM65 (**D**,**O**), c-Myc (**D**), and PAI-1 (**D**) expressions. Histograms (**E**–**O**) depict the expression comparisons of the indicated markers (*n* = 3) between the groups. CMV-PAI-1 + Control shRNA and CMV-PAI-1 + WWP1 shRNA cultures were seeded equally, allowed to grow for 5 days, and subjected to Crystal Violet staining to assess the differences in cell counts (**P**). Histogram (**Q**) shows the quantification of cell number per field (three fields per plate) (scale bar = 400 μm, 10× magnification) from (**P**) for three independent studies (*n* = 3). Data are presented as the mean ± SD, and a Student’s *T*-test was utilized for statistical comparisons. * *p* < 0.05, ** *p* < 0.01, *** *p* < 0.001, **** *p* < 0.0001.

Since we reported a strong correlation between PAI-1 (Figure 1F; Figure 2A,D; Figure 3A,D) and WWP1 (Figure 1F; Figure 2A,F; Figure 3A,F) upregulation during both UUO- and AAN-driven renal fibrogenesis as well as in human diabetic kidneys, we hypothesize that PAI-1 is a major regulator of WWP1 expression. Our screening of E3 ubiquitin ligases revealed that WWP1 is robustly induced in PAI-1-overexpressing HK-2 cells (Figure 5A–C) and primary human tubular epithelial cells (Supplementary Figure S1) relative to CMV-controls. To determine whether WWP1 is involved in PAI-1-driven tubular pathologies, we stably infected CMV-PAI-1 cultures with either control shRNA or WWP1 shRNA lentiviral constructs. WWP1 stable depletion (Figure 5D,E), indeed, mitigates the PAI-1-driven fibrotic phenotype, as marked by the downregulation of fibronectin, collagen 1, CTGF, osteopontin, and snail (Figure 5D,F–I and N). Crystal Violet-stained CMV-PAI-1 + Control shRNA and CMV-PAI-1 + WWP1 shRNA monolayers (initially seeded at similar densities and grown for 5 days) demonstrated that WWP1 depletion rescued PAI-1-induced growth defects (Figure 5P,Q), which is consistent with the reductions in p21 (Figure 5D,L) and pHistone H3 (Figure 5D,M) protein levels in our western analysis. Furthermore, WWP1 suppression in the context of sustained PAI-1 expression dramatically reduced p53 (Figure 5D,K) and pSMAD3 (Figure 5D,J) levels, both critical mediators of PAI-1-driven tubular maladaptive repair [39], compared to CMV-PAI-1 + Control shRNA cells. Indeed, activation of the transcription factors p53 and SMAD3 during renal injury is critical for fibrosis progression since proximal tubule-specific p53 knockout and global SMAD3 deficiency protects mice from renal tubular dysfunction and progressive CKD [35,40]. Therefore, tubular epithelial WWP1 upregulation is important for PAI-1 mediated tubular dysfunction, identifying WWP1 as a novel CKD-promoting entity. Moreover, stable expression of WWP1 (CMV-WWP1-GFP) in human renal tubular epithelial cells (HK-2), indeed, promotes a fibrotic response, as marked by an increase in fibronectin, collagen 1, p53, and snail expression compared to control cultures (CMV-Control) (Supplementary Figure S2), further highlighting its pro-fibrotic activity.

**Figure 6:**
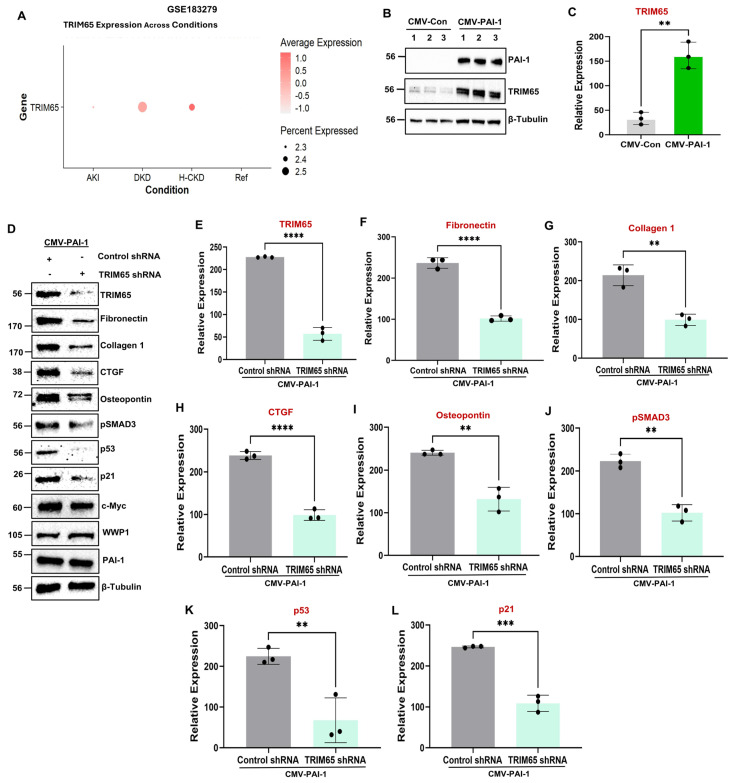
E3 ligase TRIM65 upregulation by PAI-1 is necessary for tubular dysfunction. Analysis of a single cell RNA sequencing dataset (Accession No.: GSE183279) for TRIM65 mRNA levels in human CKD patients relative to reference kidneys (healthy controls) represented as a dot plot (**A**), where the intensity of dot color (red) dictates TRIM65 expression level, and dot size represents the percentage of cells expressing a *TRIM65* gene. Immunoblot comparisons of TRIM65 protein levels between CMV-Con (reference) and CMV-PAI-1 transgenic cells lysates (**B**). The histogram in (**C**) represents the expression (mean ± SD) differences of TRIM65 protein between the groups for three independent studies (*n* = 3) in triplicate. ** *p* < 0.01. CMV-PAI-1 cells were infected with either Control shRNA (reference) or TRIM65 shRNA lentiviral particles followed by stable selection. The double transgenic cell lysates were assessed by western blotting for TRIM65 (**D**,**E**), fibronectin (**D**,**F**), collagen 1 (**D**,**G**), CTGF (**D**,**H**), osteopontin (**D**,**I**), pSMAD3 (**D**,**J**), p53 (**D**,**K**), p21 (**D**,**L**), c-Myc (**D**), WWP1 (**D**), and PAI-1 (**D**) levels. Histograms in (**E**–**L**) depict the expression differences of the indicated markers for three independent experiments (*n* = 3). Data are represented as the mean ± SD, and a Student’s *T*-test was used for statistical comparisons between groups. ** *p* < 0.01, *** *p* < 0.001, **** *p* < 0.0001.

Extensive correlations between PAI-1 (Figure 1F; Figure 2A,D; Figure 3A,D) and TRIM65 upregulation (Figure 1F; Figure 2A,G; Figure 3A,G) in the human diabetic and mouse UUO and AAN kidneys necessitates testing whether PAI-1 promotes TRIM65 induction and contributes to tubular pathologies. Indeed, TRIM65 protein expression is prominently induced in PAI-1-overexpressing HK-2 (Figure 6B,C) and RPTEC cells (Supplementary Figure S1). Moreover, genetic depletion of TRIM65 (Figure 6D,E) also abrogates the PAI-1-driven fibrotic phenotype (Figure 6D,F–I), pSMAD3 (Figure 6D,J), p53 (Figure 6D,K), and p21 (Figure 6D,L) levels, identifying a novel pathogenic role for TRIM65 induction in PAI-1-mediated tubular maladaptive repair responses. Moreover, our mechanistic study reveals that WWP1 is an important regulator of PAI-1-driven TRIM65 expression in renal tubular epithelial cells (Figure 5D,O).

### 3.4. c-Myc Is a Major Upstream Regulator of WWP1 and TRIM65 Induction and PAI-1-Driven Tubular Dysfunction

The proto-oncogene *c-Myc* regulates multiple physiological processes including cell proliferation, differentiation, and apoptosis. Hyperactivation of this transcription factor, not surprisingly, is linked to cancer progression [58]. The involvement of c-Myc in CKD progression is not well understood, although a recent study implicates c-Myc upregulation in metabolic reprogramming during CKD [41]. Whether c-Myc induction is linked to renal tubular maladaptive repair, however, requires clarification.

**Figure 7:**
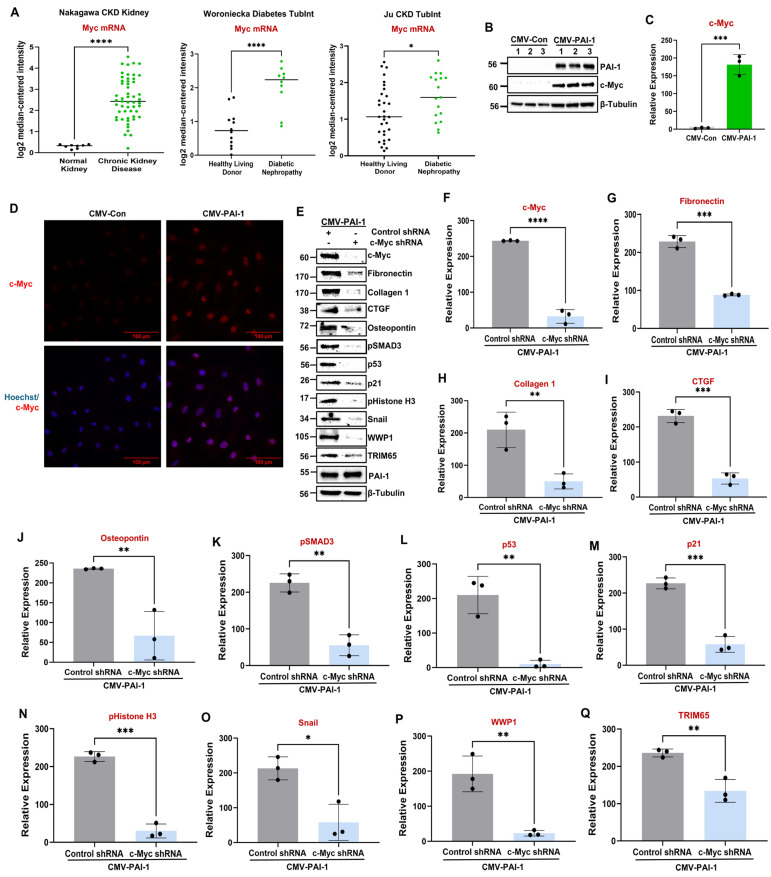
c-Myc silencing in PAI-1 stable transductants attenuates PAI-1-induced fibrotic reprogramming and WWP1 and TRIM65 induction. Assessment of Nephroseq (https://www.nephroseq.org (accessed on 5 June 2024)) renal disease datasets for c-Myc transcript levels during human CKD (Nakagawa CKD Kidney) and diabetic nephropathy (Woroniecka Diabetes TubInt, Ju CKD TubInt) progression (**A**). Data in (**A**) are represented as the median. * *p* < 0.05, **** *p* < 0.0001. CMV-Con (reference) and CMV-PAI-1 transgenic culture lysates were subjected to immunoblotting for c-Myc protein (**B**). The histogram in (**C**) represents the differences in c-Myc expression (mean ± SD) for three independent studies (*n* = 3) in triplicate. Immunofluorescence of CMV-Con and CMV-PAI-1 transgenic populations with c-Myc specific antibodies (red color) followed by Hoechst counterstaining (blue color) (**D**) (40× magnification and scale bars = 100 μm in (**D**), *n* = 3). CMV-PAI-1 cells were infected with either Control shRNA (reference) or c-Myc shRNA lentiviral particles followed by stable selection. CMV-PAI-1 + Control shRNA (reference) and CMV-PAI-1 + c-Myc shRNA double transgenic lysates were western blotted for c-Myc (**E**,**F**), fibronectin (**E**,**G**), collagen 1 (**E**,**H**), CTGF (**E**,**I**), osteopontin (**E**,**J**), pSMAD3 (**E**,**K**), p53 (**E**,**L**), p21 (**E**,**M**), pHistone H3 (**E**,**N**), snail (**E**,**O**), WWP1 (**E**,**P**), TRIM65 (**E**,**Q**), and PAI-1 (**E**) expressions. Histograms (**F**–**Q**) depict expression (mean ± SD) differences of the indicated markers (*n* = 3). A Student’s *T*-test was used for statistical analysis. * *p* < 0.05, ** *p* < 0.01, *** *p* < 0.001, **** *p* < 0.0001.

Our analysis of human renal disease datasets from Nephroseq (https://www.nephroseq.org (accessed on 5 June 2024)) revealed a significant induction in c-Myc mRNA in diseased kidney (e.g., chronic kidney disease and diabetic nephropathies) relative to normal kidneys (Figure 7A). Our analysis of human fibrotic (diabetic) and mouse models (e.g., obstruction and toxin exposure) confirms not only a significant c-Myc renal induction but also a strong association between PAI-1 (Figure 1F; Figure 2A,D; Figure 3A,D) and c-Myc (Figure 1F; Figure 2A,E; Figure 3A,E) upregulation during renal injury, suggesting a potential relationship among these entities. PAI-1, in fact, promotes a dramatic increase in c-Myc protein levels in both HK-2 (Figure 7B–D) as well as RPTEC cells (Supplementary Figure S1), and c-Myc knockdown (Figure 7E,F) mitigates PAI-1-induced fibrotic reprogramming (Figure 7E,G–O). Therefore, PAI-1 is a novel upstream regulator of c-Myc induction and subsequent tubular dysfunction. Consistent with the previous studies demonstrating that c-Myc is a direct transcriptional activator of WWP1 during tumorigenesis [21], our western analysis revealed that c-Myc depletion in PAI-1 transductants also dramatically decreased WWP1 (Figure 7E,P) and TRIM65 protein levels (Figure 7E,Q). Collectively, these data indicate that PAI-1-driven WWP1 and TRIM65 induction requires c-Myc activation during tubular dysfunction.

### 3.5. PAI-1-Induced Repression of the Antifibrotic BMP-7/SMAD1/5 Pathway Is Causatively Linked to Tubular Dysfunction

**Figure 8:**
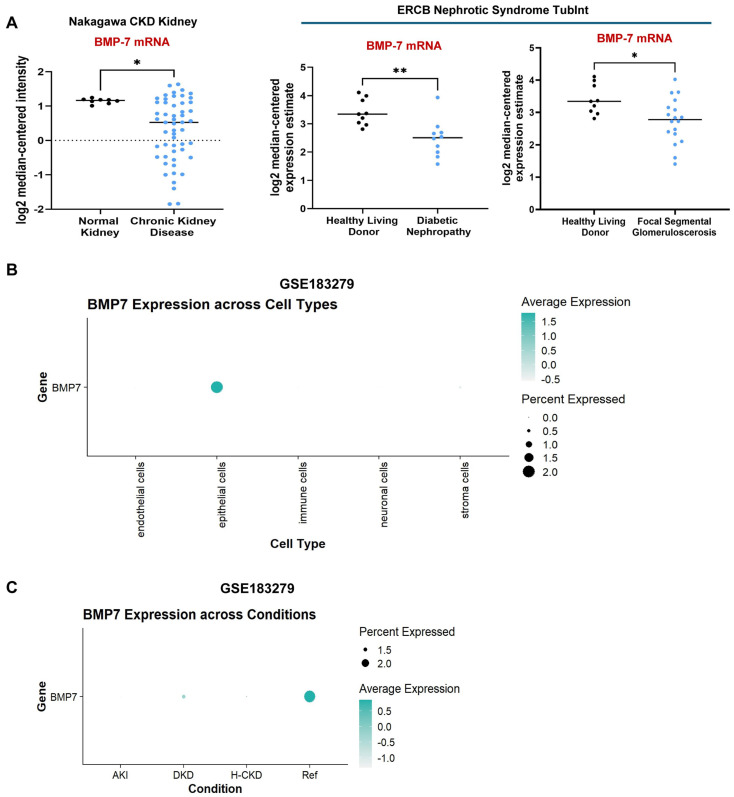
Renal expression of BMP-7 is frequently lost during human chronic renal injury. Assessment of BMP-7 mRNA levels in human CKD (Nakagawa CKD Kidney), diabetic, and focal segmental glomerulosclerosis (ERCB Nephrotic Syndrome TubInt) renal disease datasets available in the Nephroseq (https://www.nephroseq.org (accessed on 5 June 2024)) database (**A**). Data in (**A**) are presented as the median. * *p* < 0.05, ** *p* < 0.01. Evaluation of a recent single cell RNA sequencing dataset (Accession No.: GSE183279) for the renal compartment-specific expression of BMP-7 transcripts (**B**) and its differential expression (**C**) in human AKI, DKD, and hypertensive CKD (H-CKD) patients compared to healthy reference kidneys, represented as dot plots, where the intensity of dot color (sea green) dictates BMP-7 expression level, and dot size represents the percentage of cells expressing a *BMP-7* gene.

**Figure 9:**
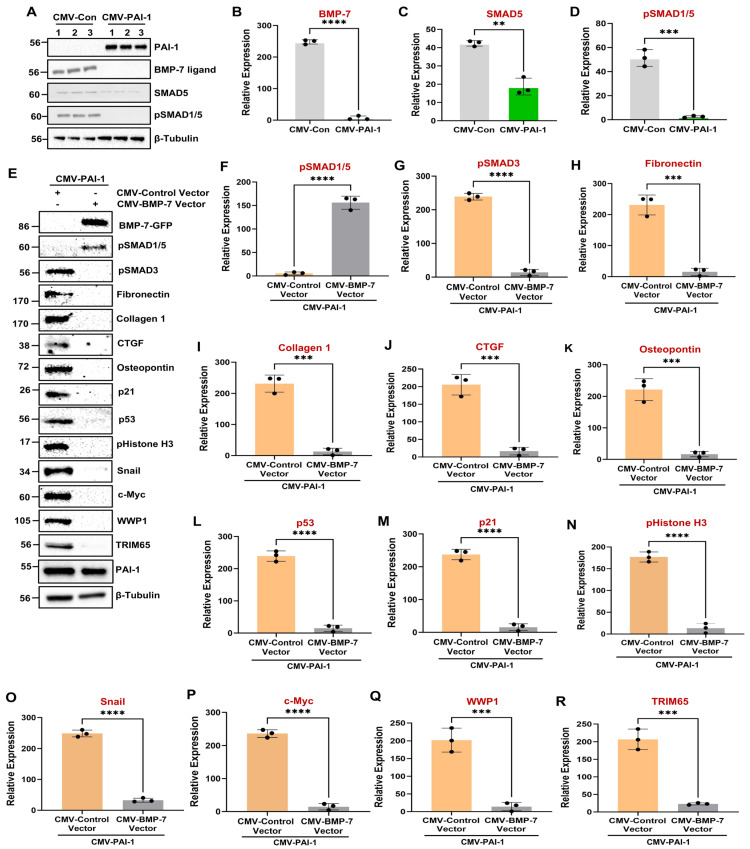
Tubular PAI-1 upregulation impairs the BMP-7/SMAD1/5 signaling network, and rescue of BMP-7 expression mitigates PAI-1-induced fibrogenesis and c-Myc, WWP1, and TRIM65 upregulation. CMV-Con and CMV-PAI-1 cell lysates were subjected to western blot analysis for the expression of BMP-7, SMAD5, and pSMAD1/5 (**A**). Histograms (**B**–**D**) depict the expression comparisons of BMP-7 (**B**), SMAD5 (**C**), and pSMAD1/5 (**D**) between CMV-Con (reference) and CMV-PAI-1 cell populations in three independent experiments (*n* = 3) in triplicate. ** *p* < 0.01, *** *p* < 0.001, **** *p* < 0.0001. PAI-1-overexpressing cells were infected with either CMV-Control (reference) or CMV-BMP-7 expressing lentiviral particles prior to stable selection. CMV-BMP-7 constructs have a GFP tag at their C terminal end. Double transgenic cell lysates were extracted and immunoblotted for GFP (**E**), pSMAD1/5 (**E**,**F**), pSMAD3 (**E**,**G**), fibronectin (**E**,**H**), collagen 1 (**E**,**I**), CTGF (**E**,**J**), osteopontin (**E**,**K**), p53 (**E**,**L**), p21 (**E**,**M**), pHistone H3 (**E**,**N**), snail (**E**,**O**), c-Myc (**E**,**P**), WWP1 (**E**,**Q**), TRIM65 (**E**,**R**), PAI-1 (**E**), and β-tubulin (**E**) levels. Histograms (**F**–**R**) depict expression differences of the indicated markers between CMV-PAI-1 + CMV-Control Vector (reference) and CMV-PAI-1 + CMV-BMP-7 Vector cells for three independent experiments (*n* = 3). All data are presented as the mean ± SD, and a Student’s *T*-test was used for statistical comparison between the indicated groups. *** *p* < 0.001, **** *p* < 0.0001.

BMP-7 is a member of the TGF-β superfamily of ligands that binds to its cognate receptors to initiate SMAD1/5 phosphorylation which, in turn, antagonizes profibrotic SMAD3 signaling triggered by TGF-β1 [42,47]. The expression of BMP-7 and SMAD5, as well as SMAD1/5 phosphorylation, is markedly downregulated in both UUO (Figure 2A,H–J) and AAN (Figure 3A,H–J) mouse models. While the mRNA expression of BMP-7 is particularly enriched in the renal epithelial compartment (Figure 8B), the levels are downregulated during human CKD progression (Figure 8A,C), consistent with previous observations [46,59]. Administration of recombinant BMP-7 protein during renal injury, on the other hand, mitigates fibrosis and improves kidney health, demonstrating its utility as a potent antifibrotic target [43,44,45,60]. Even though BMP-7 is an attractive antifibrotic target, upstream regulation of BMP-7 repression during the progression of renal disease, however, is not well understood. In this regard, whether PAI-1 represses BMP-7-SMAD1/5 pathways during CKD progression has not been tested. Immunoblotting of CMV-Con and CMV-PAI-1 cell extracts revealed that PAI-1 overexpression not only dramatically decreased BMP-7 ligand levels (Figure 9A,B) and SMAD1/5 phosphorylation (Figure 9A,D) but also repressed total SMAD5 protein levels in HK-2 cells (Figure 9A,C). Therefore, PAI-1 is a novel repressor of the renal tubular BMP-7/SMAD1/5 signaling axis, which is further confirmed in RPTEC cells (Supplementary Figure S1). To determine whether BMP-7 loss of expression is causatively linked to PAI-1-induced maladaptive repair, CMV-PAI-1 cells were infected with either CMV-Control Vector or CMV-BMP-7 lentiviral constructs prior to stable selection. Western blot analysis confirmed that restoration of BMP-7 expression in CMV-PAI-1 cells (Figure 9E) attenuates PAI-1-driven fibrotic response, as evident by major reductions in pSMAD3 (Figure 9E,G), fibronectin (Figure 9E, H), collagen 1 (Figure 9E,I), CTGF (Figure 9E,J), osteopontin (Figure 9E,K), p53 (Figure 9E, L), p21 (Figure 9E,M), pHistone H3 (Figure 9E,N), and snail (Figure 9E,O) levels compared to CMV-PAI-1 + CMV-Control Vector cells. As anticipated, BMP-7 restoration triggers pSMAD1/5 phosphorylation (Figure 9E,F). These findings indicate PAI-1-induced BMP-7 loss as a promoter of tubular dysfunction.

**Figure 10:**
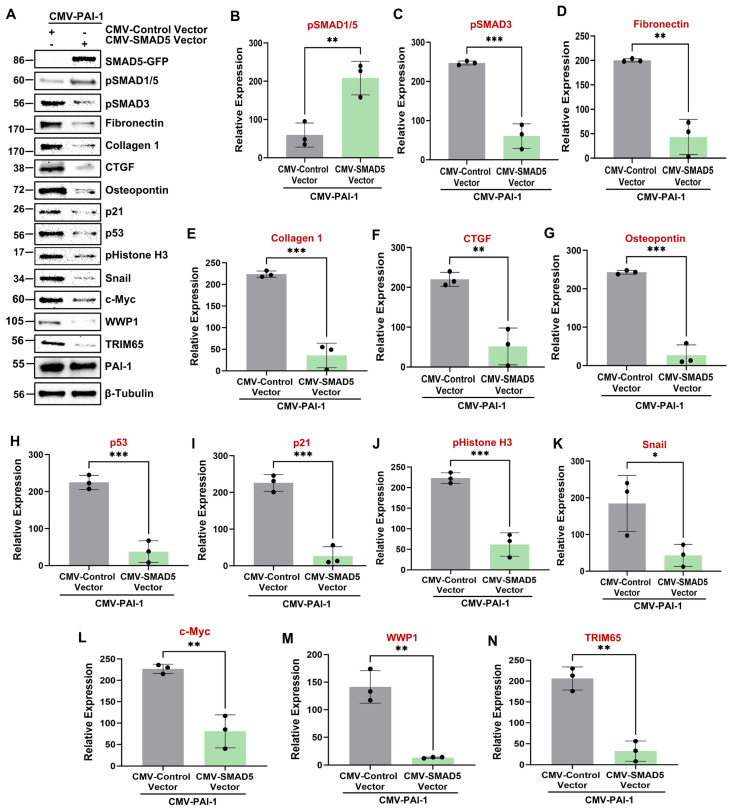
Restoration of SMAD5 expression attenuates PAI-1-driven fibrogenic responses as well as c-Myc, WWP1, and TRIM65 induction. CMV-PAI-1 cells were stably infected with either CMV-Control (reference) or CMV-SMAD5 lentiviral particles followed by stable selection. CMV-SMAD5 constructs are linked to a GFP Tag at their C terminal end. Immunoblot analysis of double transgenic lysates for GFP (**A**), pSMAD1/5 (**A**,**B**), pSMAD3 (**A**,**C**), fibronectin (**A**,**D**), collagen 1 (**A**,**E**), CTGF (**A**,**F**), osteopontin (**A**,**G**), p53 (**A**,**H**), p21 (**A**,**I**), pHistone H3 (**A**,**J**), snail (**A**,**K**), c-Myc (**A**,**L**), WWP1 (**A**,**M**), TRIM65 (**A**,**N**), PAI-1 (**A**), and β-tubulin (**A**) proteins. Histograms (**B**–**N**) depict expression differences for indicated proteins between CMV-PAI-1 + CMV-Control Vector (reference) and CMV-PAI-1 + CMV-SMAD5 Vector lysates in three independent experiments (*n* = 3). Data are presented as the mean ± SD, and a Student’s *T*-test was utilized for statistical comparisons. * *p* < 0.05, ** *p* < 0.01, *** *p* < 0.001.

SMAD1/5 are direct downstream targets of the BMP-7 pathway by receptor-mediated phosphorylation [47]. Since PAI-1 is a novel repressor of SMAD5 protein levels, we tested whether SMAD5 repression is linked to tubular dysfunction. Ectopic restoration of SMAD5 expression (Figure 10A) in CMV-PAI-1 transgenic cells via lentiviral infection with CMV driven SMAD5 expression constructs significantly attenuates PAI-1 mediated tubular dysfunctional phenotype compared to vector-transduced double transgenic controls (CMV-PAI-1+CMV-vector) (Figure 10A–K). These data identify PAI-1 as a major repressor of BMP-7 signaling cascade and that disabling of BMP-7/SMAD5 signaling axis is linked to PAI-1-driven tubular maladaptive repair.

### 3.6. BMP-7 and SMAD5 Are Upstream Regulators of PAI-1-Driven c-Myc, WWP1, and TRIM65 Upregulation

The pathologic links among renal PAI-1 upregulation, BMP-7/SMAD1/5 repression, and c-Myc, WWP1, and TRIM65 induction are currently unclear. Intriguingly, BMP-7 repression downstream of PAI-1 promotes c-Myc, WWP1, and TRIM65 induction and renal tubular pathogenesis since rescue of BMP-7 expression in PAI-1 stable transductants abrogates PAI-1-driven c-Myc (Figure 9E,P), WWP1 (Figure 9E,Q), and TRIM65 (Figure 9E,R) expression. Similarly, restoration of SMAD5 levels (which is otherwise downregulated by PAI-1) also leads to a significant reduction in c-Myc (Figure 10A,L), WWP1 (Figure 10A,M), and TRIM65 (Figure 10A,N) expression downstream of PAI-1 overexpression, suggesting that SMAD5 downregulation is a critical upstream regulator of c-Myc, WWP1 and TRIM65 induction by PAI-1. Repression of BMP-7 and SMAD5 signaling in the UUO (Figure 2A,H-J) and AAN (Figure 3A,H-J) kidney injury models correlates with increased c-Myc (Figure 2A,E; Figure 3A,E), WWP1 (Figure 2A,F; Figure 3A,F) and TRIM65 (Figure 2A,G; Figure 3A,G) expression in mice, further highlighting the potential causative relationship among these entities during CKD progression. These data highlight a previously unknown pathogenic relationship between BMP-7/SMAD5 downregulation and induction of c-Myc, WWP1 and TRIM65 proteins during progressive renal injury.

## 4. Discussion

Our study uncovers several novel mechanistic insights into tubular maladaptive repair and renal fibrosis progression. We identify WWP1, an E3 ubiquitin ligase, as a previously unrecognized and highly upregulated factor in tubular epithelium during renal injury in both human CKD specimens (Figure 1) and murine UUO (Figure 2) and AAN (Figure 3) kidneys. Importantly, WWP1 expression correlates with CKD progression in humans (Figure 1), highlighting its potential clinical relevance. While proteasome inhibition is known to confer renoprotection [18], the specific contributions of E3 ligases in CKD pathogenesis remain underexplored. Our findings establish WWP1 as a novel mediator of tubular epithelial dysfunction and fibrogenesis. PAI-1 is robustly upregulated in renal tubules across injury models and promotes dysfunction via p53- and pSMAD3-dependent pathways [27,36,39]. We further identify PAI-1 as a novel upstream inducer of WWP1 (Figure 5; Supplementary Figure S1). Silencing WWP1 reverses PAI-1-driven maladaptive repair by reducing epithelial differentiation, relieving cell cycle inhibition, and decreasing extracellular matrix and fibrotic marker expression and pSMAD3/p53 signaling (Figure 5). To our knowledge, this is the first demonstration of PAI-1 regulating WWP1 in any pathological context. Moreover, WWP1 ectopic expression in renal tubular epithelial cells alone is sufficient to induce a fibrotic response (Supplementary Figure S2).

Furthermore, our study identifies TRIM65 as another novel E3 ligase regulated by PAI-1. Although global TRIM65 knockout mice are protected from UUO and folic acid-driven renal fibrosis [26], its precise role in tubular pathogenesis is unclear. PAI-1 robustly induces TRIM65 expression (Figure 6; Supplementary Figure S1), and stable silencing of TRIM65 inhibits PAI-1-driven fibrotic reprogramming (Figure 6), linking TRIM65 upregulation to tubular pathogenesis. While WWP1 depletion leads to the attenuation of PAI-1-driven TRIM65 expression (Figure 5), repression of TRIM65 in PAI-1 transductants does not impact WWP1 levels (Figure 6). Therefore, we also uncover WWP1 as an upstream regulator of TRIM65 during tubular dysfunction. Our current findings reveal that silencing either WWP1 or TRIM65 mitigates p53 levels and SMAD3 phosphorylation (Figure 5 and Figure 6), two key transcriptional regulators of renal tubular dysfunction and fibrosis [35,40]. WWP1 and TRIM65 upregulation is linked to PTEN and PPM1A ubiquitination, respectively [21,25]. Indeed, PPM1A and PTEN levels are dramatically decreased during kidney fibrosis originating from various etiologies [50,51], and tubular PPM1A and PTEN depletion alone triggers epithelial dedifferentiation, growth inhibition, and fibrosis via SMAD3 and p53 activation [50,51]. Therefore, WWP1- and TRIM65-mediated regulation of p53 and pSMAD3 could be linked to PTEN and PPM1A ubiquitination, leading to fibrotic maladaptive repair and fibrosis.

Previous studies demonstrated that BMP-7 is a potent CKD therapeutic target as the administration of recombinant BMP-7 mitigates UUO-driven fibrogenesis and improves renal health [43,44,45,60]. Here, we identify PAI-1 as a novel repressor of the BMP-7/SMAD1/5 axis. BMP-7 transcript levels are reduced in fibrotic human kidneys (Figure 8), consistent with the findings in CKD mouse models [46,59]. We demonstrate that PAI-1 represses BMP-7 protein levels, inhibits SMAD1/5 phosphorylation, and reduces total SMAD5 expression, highlighting a multilevel disruption of this signaling cascade (Figure 9; Supplementary Figure S1). Restoration of BMP-7 (Figure 9) or SMAD5 (Figure 10) reverses PAI-1-induced fibrotic reprogramming and suppresses c-Myc, WWP1, TRIM65, and downstream pSMAD3/p53 activation. Regardless of the genetic manipulation (either knockdown of c-Myc, WWP1, and TRIM65 or overexpression of BMP-7 and SMAD5), PAI-1 levels in the double transgenic population remain comparable (Figure 5, Figure 6, Figure 7, Figure 9 and Figure 10). Therefore, we define a previously unrecognized PAI-1–BMP-7/SMAD5–c-Myc–WWP1–TRIM65 regulatory hierarchy in tubular dysfunction. Future studies will determine the mechanism by which PAI-1 downregulates the BMP-7 ligand and how loss of BMP-7/SMAD5 signaling triggers c-Myc activation.

## 5. Conclusions

**Figure 11:**
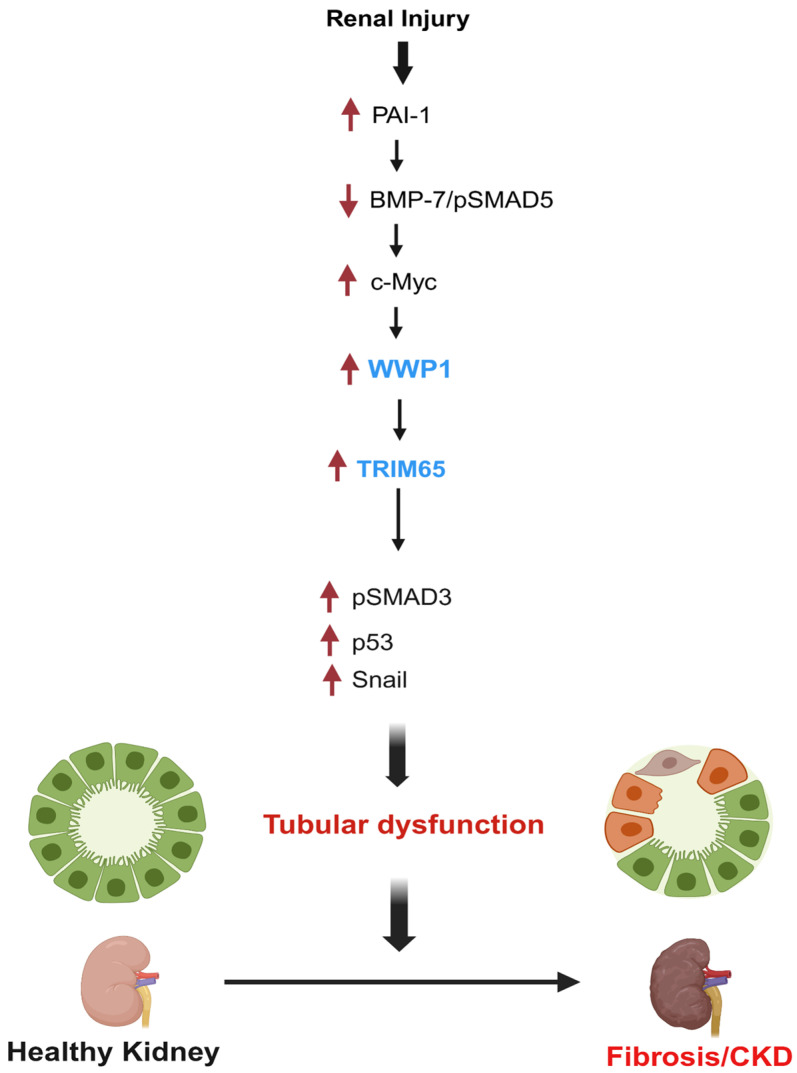
Model of WWP1 and TRIM65 involvement in renal tubular dysfunction and fibrosis (created with BioRender: https://www.biorender.com/). PAI-1 strongly induces the expression of the E3 ubiquitin ligases WWP1 and TRIM65, which are causatively linked to maladaptive tubular repair. Sustained renal epithelial PAI-1 induction represses BMP-7 expression, leading to tubular dysfunction. PAI-1-mediated suppression of the anti-fibrotic BMP-7–SMAD5 signaling axis triggers upregulation of the transcription factor c-Myc, which in turn drives WWP1 and TRIM65 induction, subsequent SMAD3 and p53 activation, and renal maladaptive repair.

In summary, we identify WWP1 and TRIM65 as previously unrecognized mediators of tubular dysfunction. Mechanistically, PAI-1 suppresses the BMP-7/SMAD5 axis, thereby, triggering tubular fibrosis through activation of the c-Myc–WWP1–TRIM65 cascade (Figure 11). Our findings establish a novel paradigm in which PAI-1 acts as a dual-function mediator of renal fibrosis by suppressing regenerative pathways (BMP-7/SMAD5 axis) and activating profibrotic cascades (WWP1, c-Myc, p53, and SMAD3). Therefore, PAI-1 can be a compelling therapeutic target to restore the balance between pro- and anti-fibrotic signals during maladaptive repair. Inhibition of WWP1 could also offer a promising strategy to halt renal fibrosis progression.

## Data Availability

All data derived from this work are included in this manuscript. Data of all human renal transcriptomics datasets for respective genes are available in the Nephroseq database (https://www.nephroseq.org (accessed on 5 June 2024)) under the respective dataset identifiers (ERCB Nephrotic Syndrome TubInt, ERCB Lupus TubInt, Nakagawa CKD Kidney, Woroniecka Diabetes TubInt, and Ju CKD TubInt). Data for the renal single cell RNA sequencing atlas are accessible from the Gene Expression Omnibus (https://www.ncbi.nlm.nih.gov/geo/ (accessed on 4 February 2025)) under Accession No.: GSE183279.

## Supplementary Materials

The following supporting information can be downloaded at: https://www.mdpi.com/article/10.3390/biom16030373/s1, Figure S1: PAI-1 induction in primary human renal tubular epithelial cells drives fibrotic reprogramming, upregulation of c-Myc, WWP1, and TRIM65, and repression of the BMP-7/SMAD5 signaling axis; Figure S2: Ectopic expression of WWP1 in renal tubular epithelial cells is sufficient to promote a fibrotic response; Figure S3: Unprocessed original western blot images.

## Abbreviations

The following abbreviations are used in this manuscript: AA: aristolochic acid, AAN: AA nephropathy, BMP-7: bone morphogenetic protein-7, BSA: bovine serum albumin, CKD: chronic kidney disease, CCN2/CTGF: cellular communication network factor 2/connective tissue growth factor, CMV: cytomegalovirus, c-Myc: cellular Myc, P^21^: cyclin-dependent kinase inhibitor 1, r: correlation coefficient, DMSO: dimethylsulfoxide, DMEM: Dulbecco’s modified eagle medium, DN: diabetic nephropathy, ESRD: end-stage renal disease, ECM: extracellular matrix, ERCB: European renal cDNA bank, FBS: fetal bovine serum, FSGS: focal segmental glomerulosclerosis, GEO: Gene Expression Omnibus, GFP: green fluorescent protein, GFR: glomerular filtration rate, HK-2: human kidney 2, HRP: horseradish peroxidase, mRNA: messenger RNA, PAI-1: plasminogen activator inhibitor type-1, PPM1A: protein phosphatase magnesium/manganese dependent 1A, PTEN: phosphate tensin homolog on chromosome 10, PBS: phosphate-buffered saline, PAGE: polyacrylamide gel electrophoresis, pSMAD3: phospho-SMAD3, pHistone H3: phospho-histone H3, pSMAD1/5: phospho-SMAD1/5, PCA: principal component analysis, SDS: Sodium dodecyl sulfate, SD: standard deviation, SMAD: mothers against decapentaplegic, shRNA: short hairpin RNA, SASP: senescence-like secretory phenotype, TGF-β1: transforming growth factor-β1, TRIM65: tripartite motif-containing protein 65, UUO: unilateral ureteral obstruction, UMAP: uniform manifold approximation and projection, UPS: ubiquitin proteasome system, WWP1: WW domain-containing E3 ubiquitin protein ligase 1.
