# Supplementary material for: Involvement of c-Myc/WWP1/TRIM65 Axis in Renal Fibrosis
Source: Biomolecules. 2026 Mar 2;16(3):373. doi: 10.3390/biom16030373 (PMC13024234; doi:10.3390/biom16030373)
Supplement: Supplementary file 1 [file biomolecules-16-00373-s001.zip › biomolecules-4071032-supplementary.pdf]

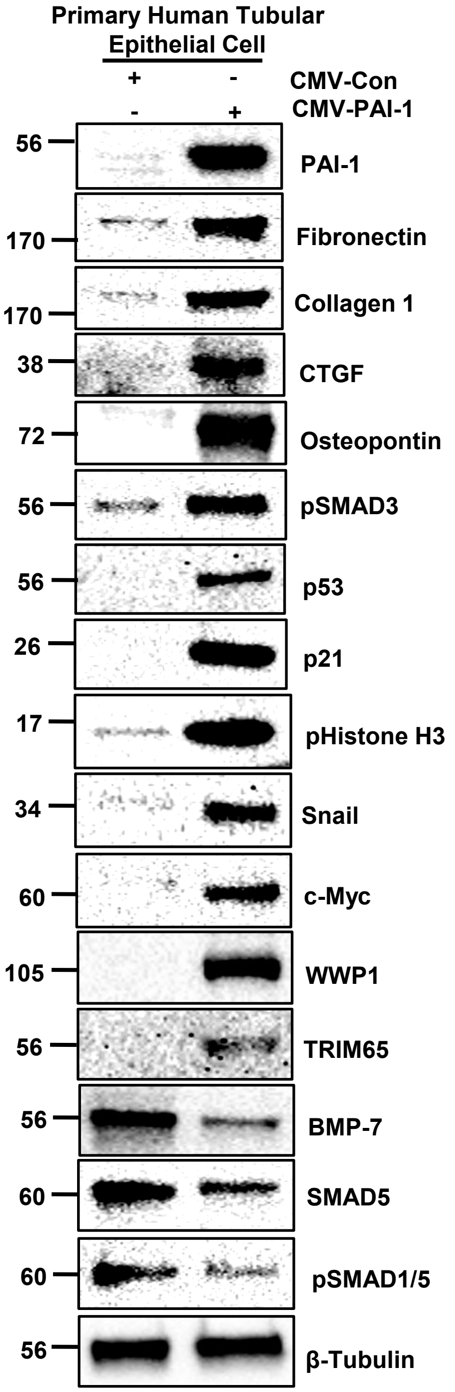

Supplementary Figure S1. PAI-1 induction in primary human renal tubular epithelial cells drives fibrotic reprogramming, upregulation of c-Myc, WWP1, and TRIM65, and repression of the BMP-7/SMAD5 signaling axis. Lysates from RPTEC cells expressing either the CMV-Control vector or the CMV-PAI-1 vector were analyzed by western blot for PAI-1, fibronectin, collagen I, CTGF, osteopontin, pSMAD3, p53, p21, pHistone H3, Snail, c-Myc, WWP1, TRIM65, BMP-7, SMAD5, pSMAD1/5, and β-tubulin (loading control).

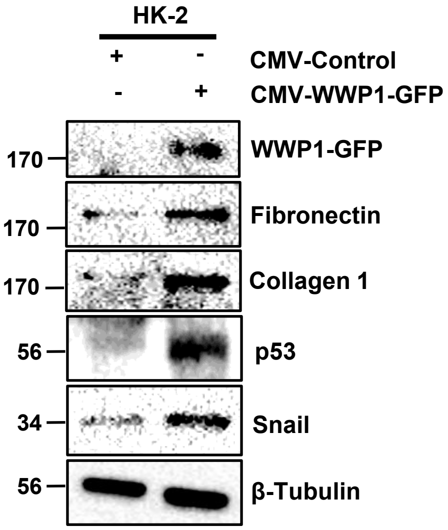

Supplementary Figure S2. Ectopic expression of WWP1 in renal tubular epithelial cells is sufficient to promote a fibrotic response. Cellular extracts of HK-2 cells expressing either the CMV-Control vector or the CMV-WWP1-GFP vector were subjected to immunoblot analysis for GFP, fibronectin, collagen 1, p53, snail, β-tubulin (loading control).

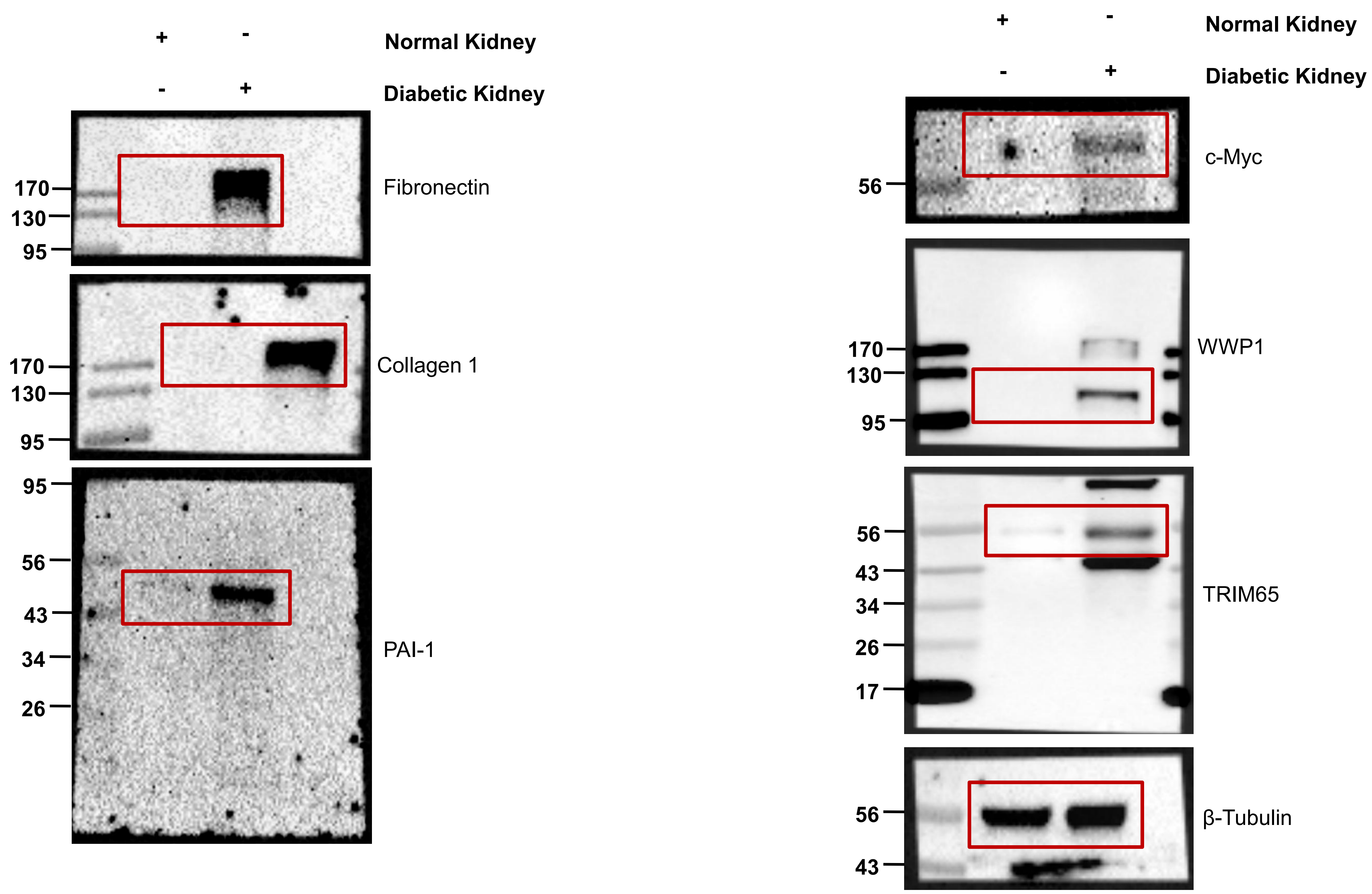

Supplementary Figure S3: Unprocessed original images of blots shown in Figure 1F

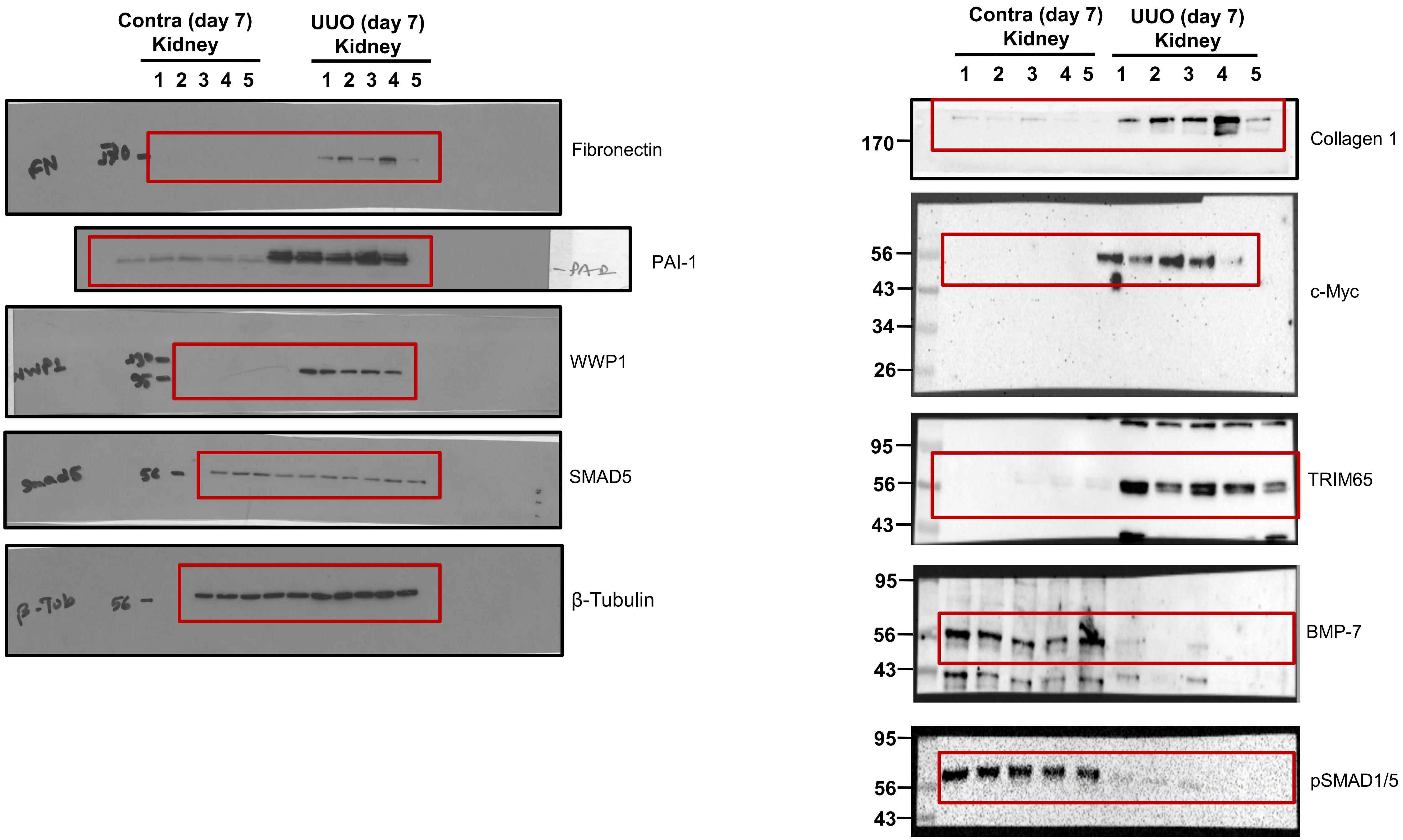

Supplementary Figure S3: Unprocessed original images of blots shown in Figure 2A

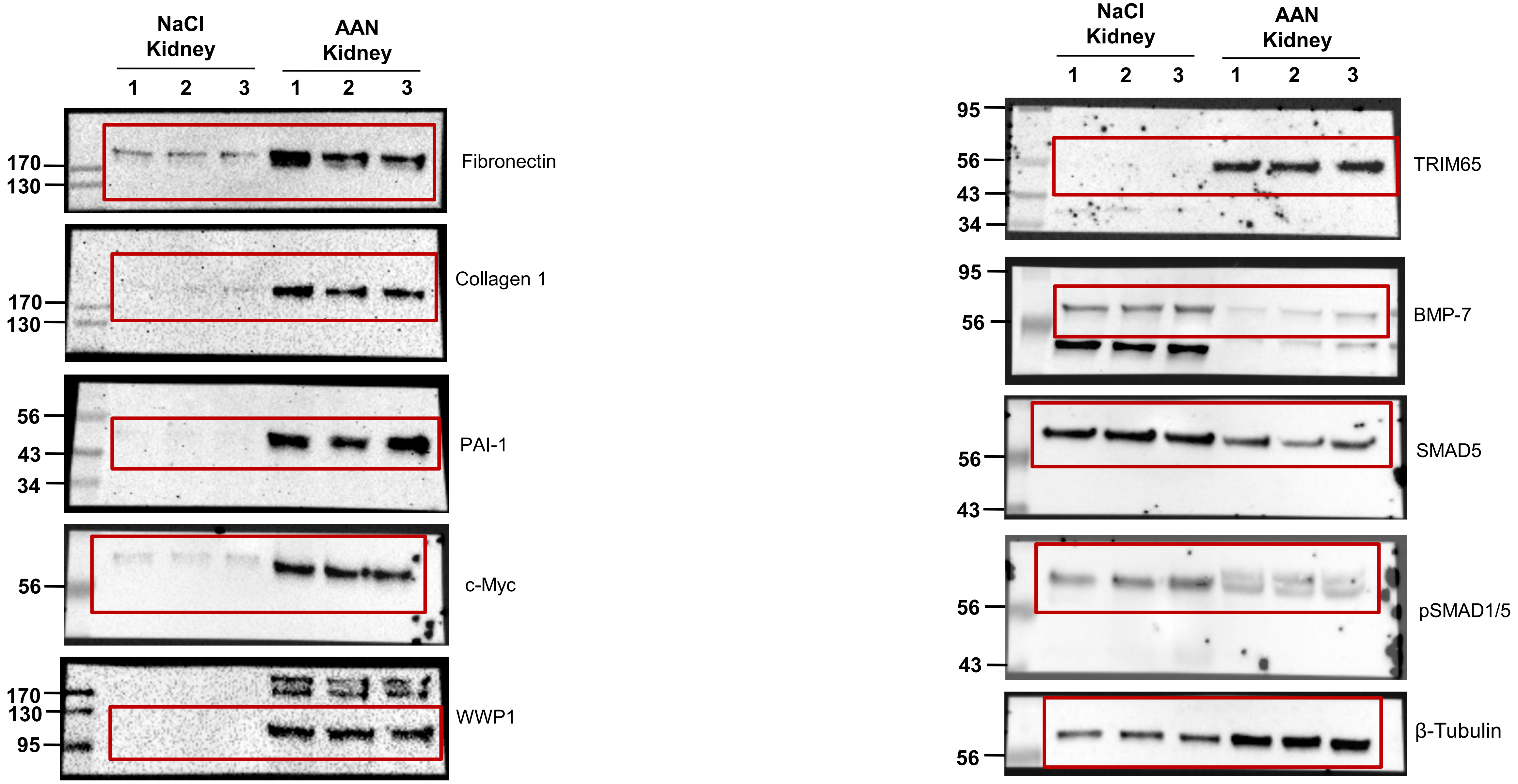

Supplementary Figure S3: Unprocessed original images of blots shown in Figure 3A

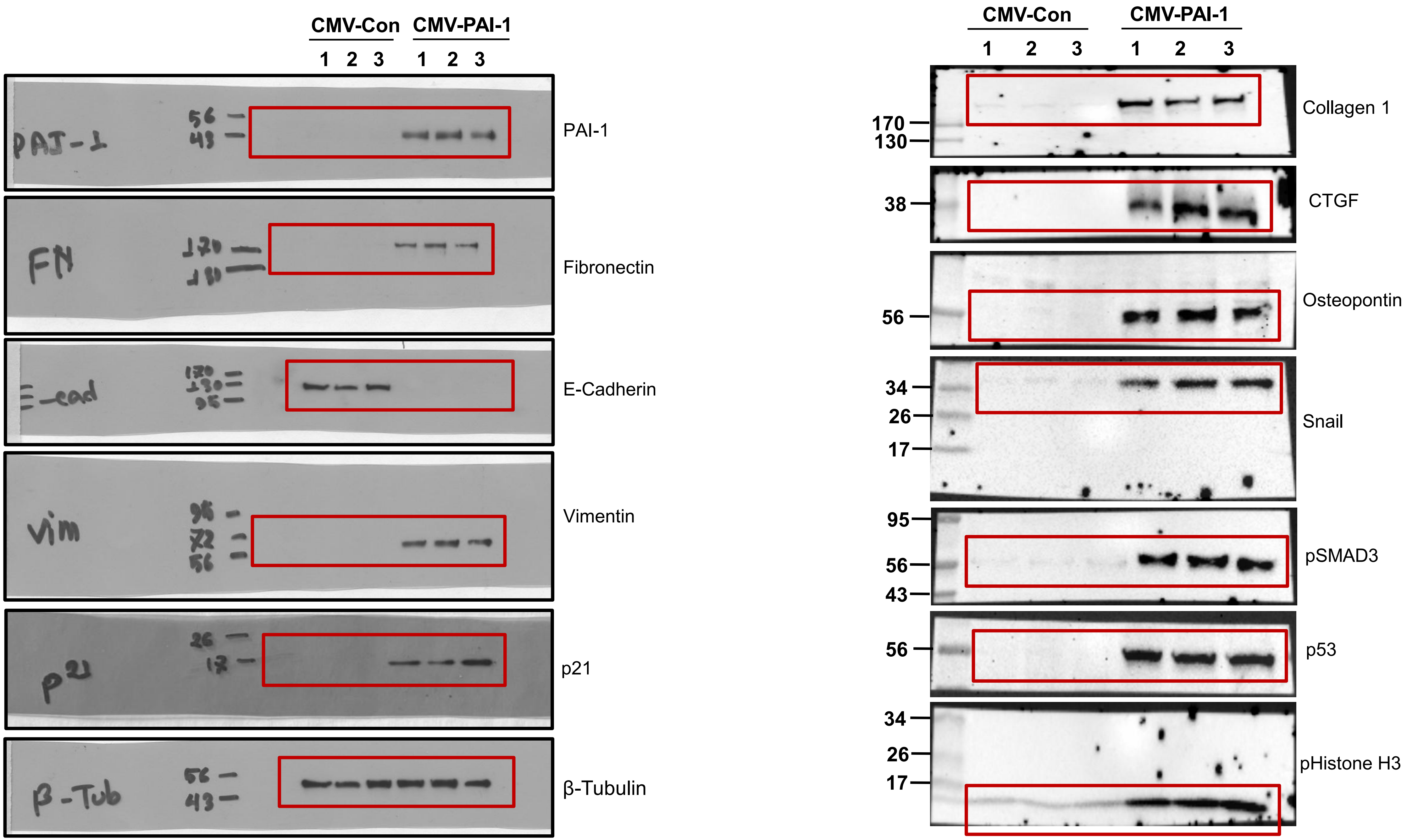

Supplementary Figure S3: Unprocessed original images of blots shown in Figure 4B

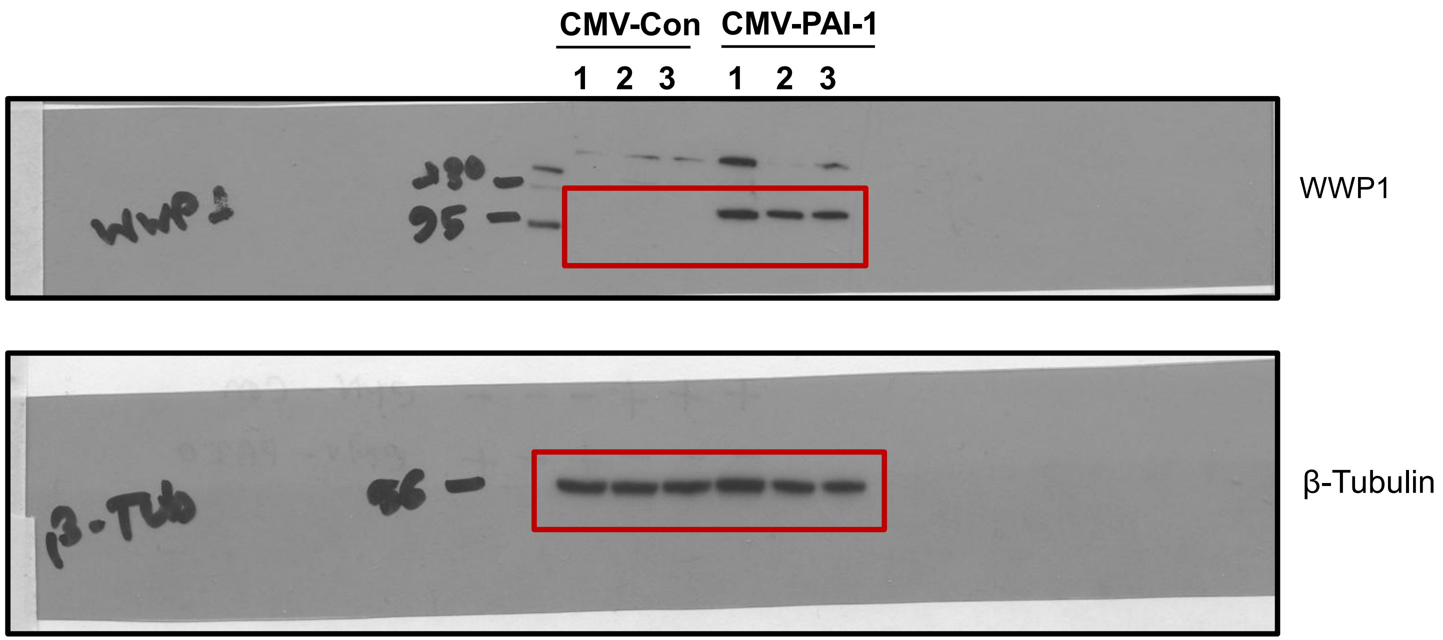

Supplementary Figure S3: Unprocessed original images of blots shown in Figure 5A

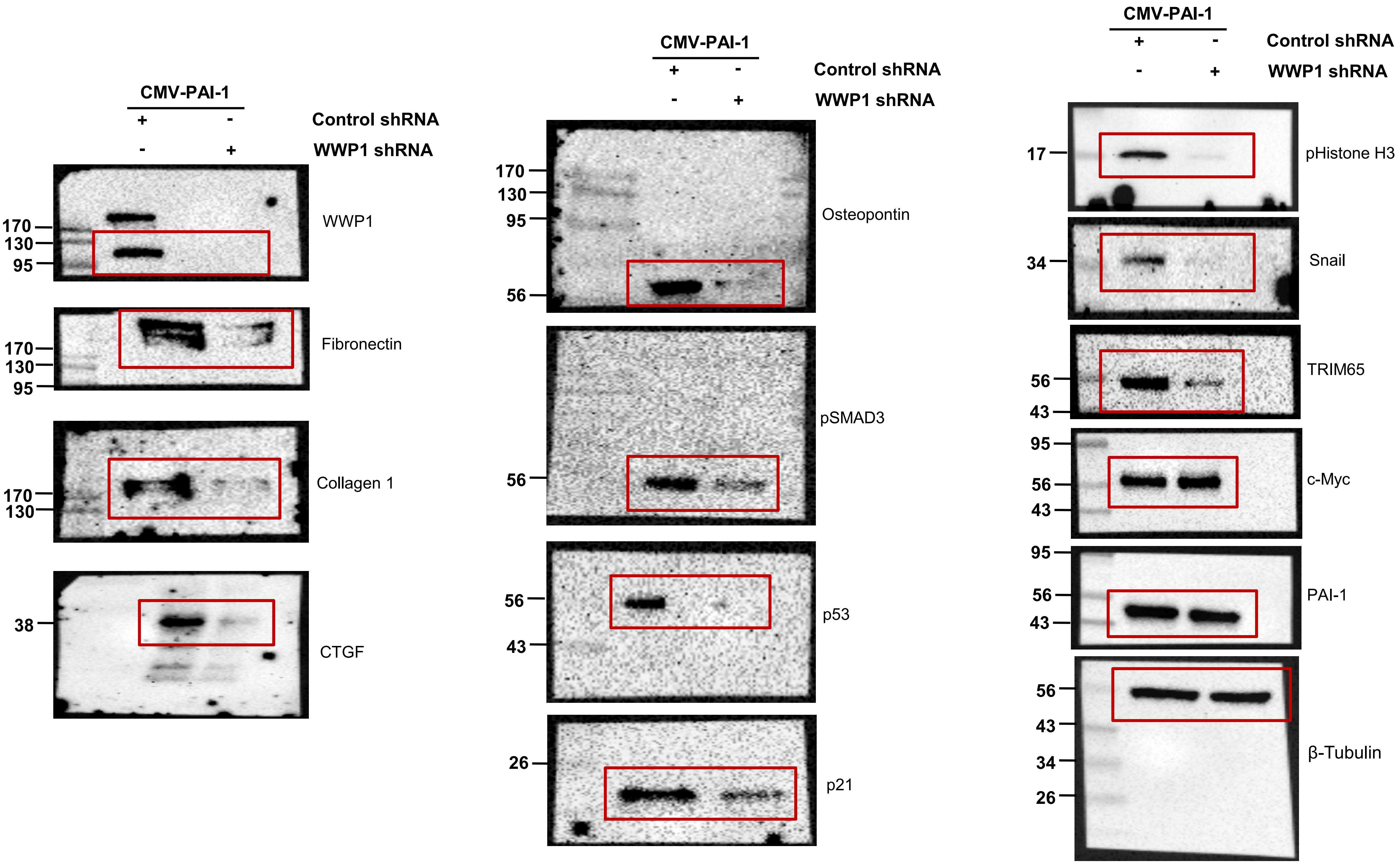

Supplementary Figure S3: Unprocessed original images of blots shown in Figure 5D

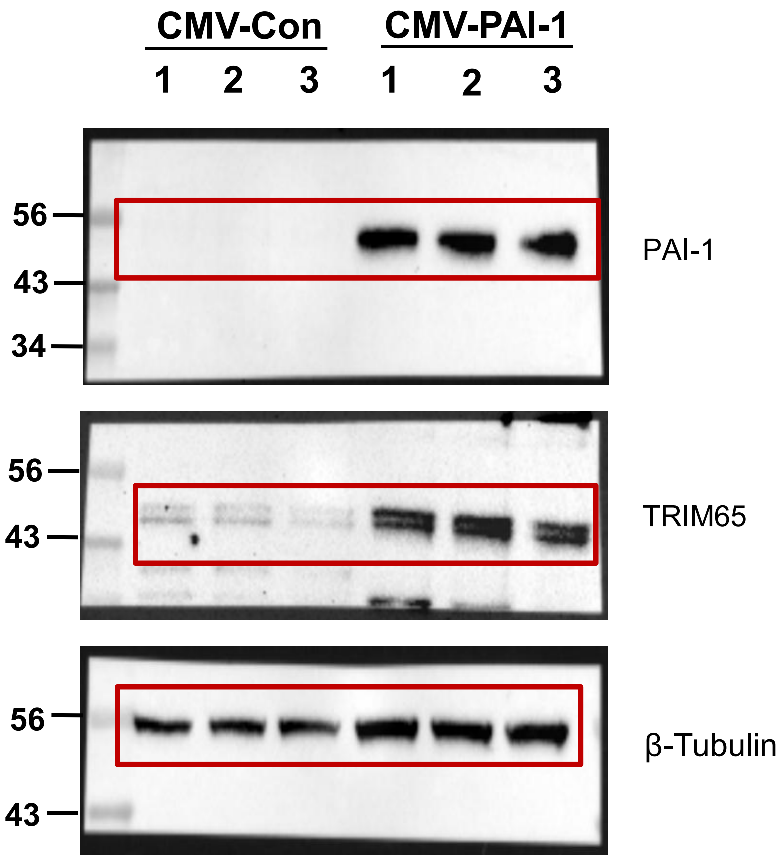

Supplementary Figure S3: Unprocessed original images of blots shown in Figure 6B

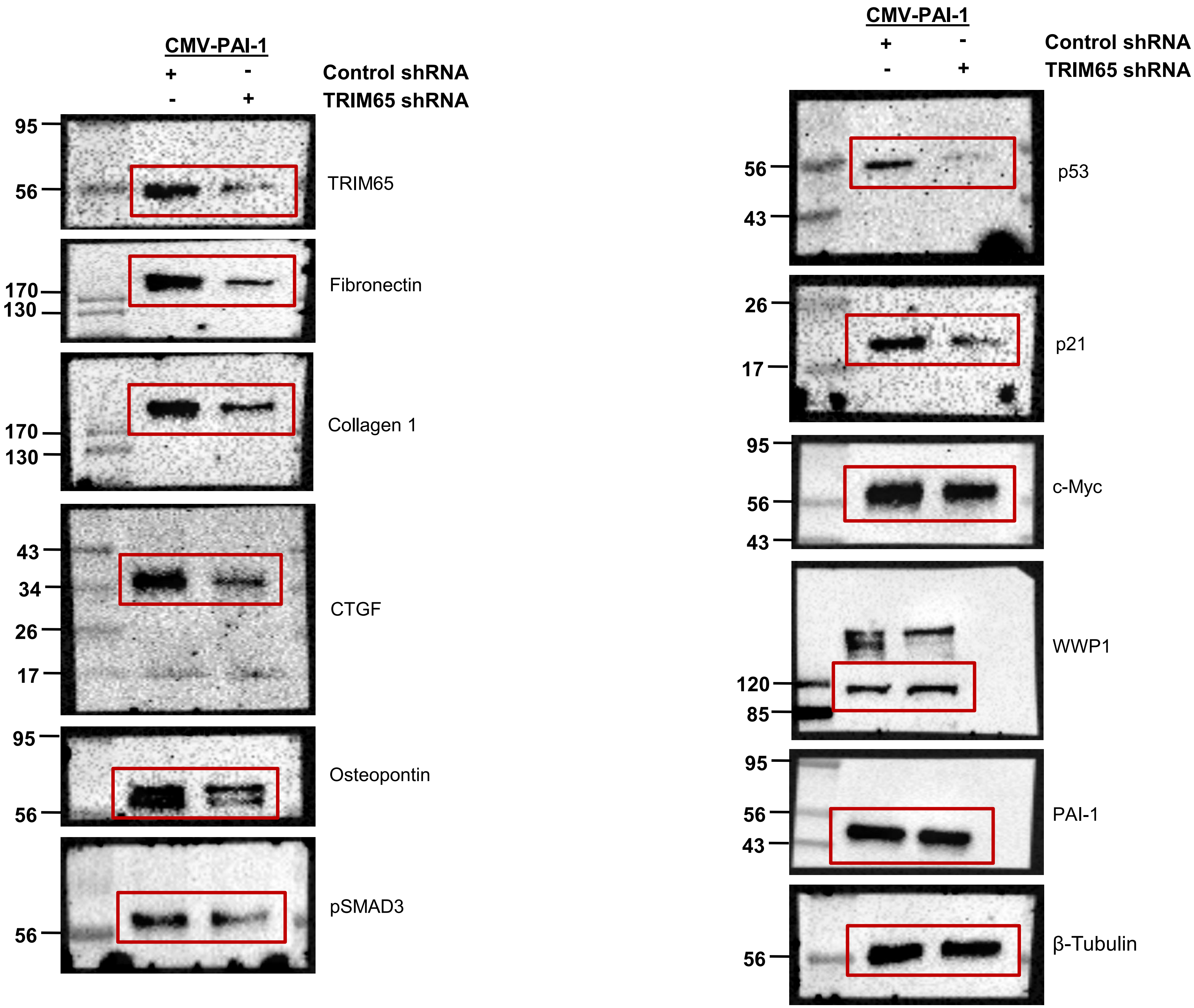

Supplementary Figure S3: Unprocessed original images of blots shown in Figure 6D

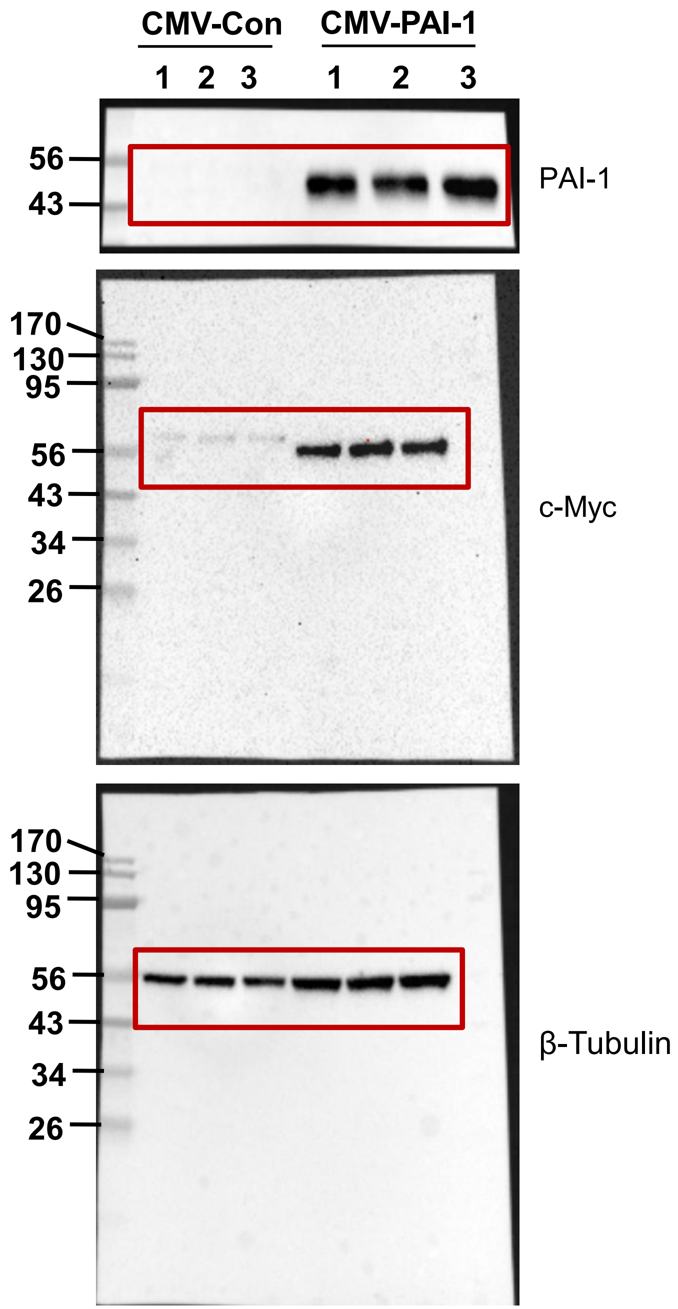

Supplementary Figure S3: Unprocessed original images of blots shown in Figure 7B

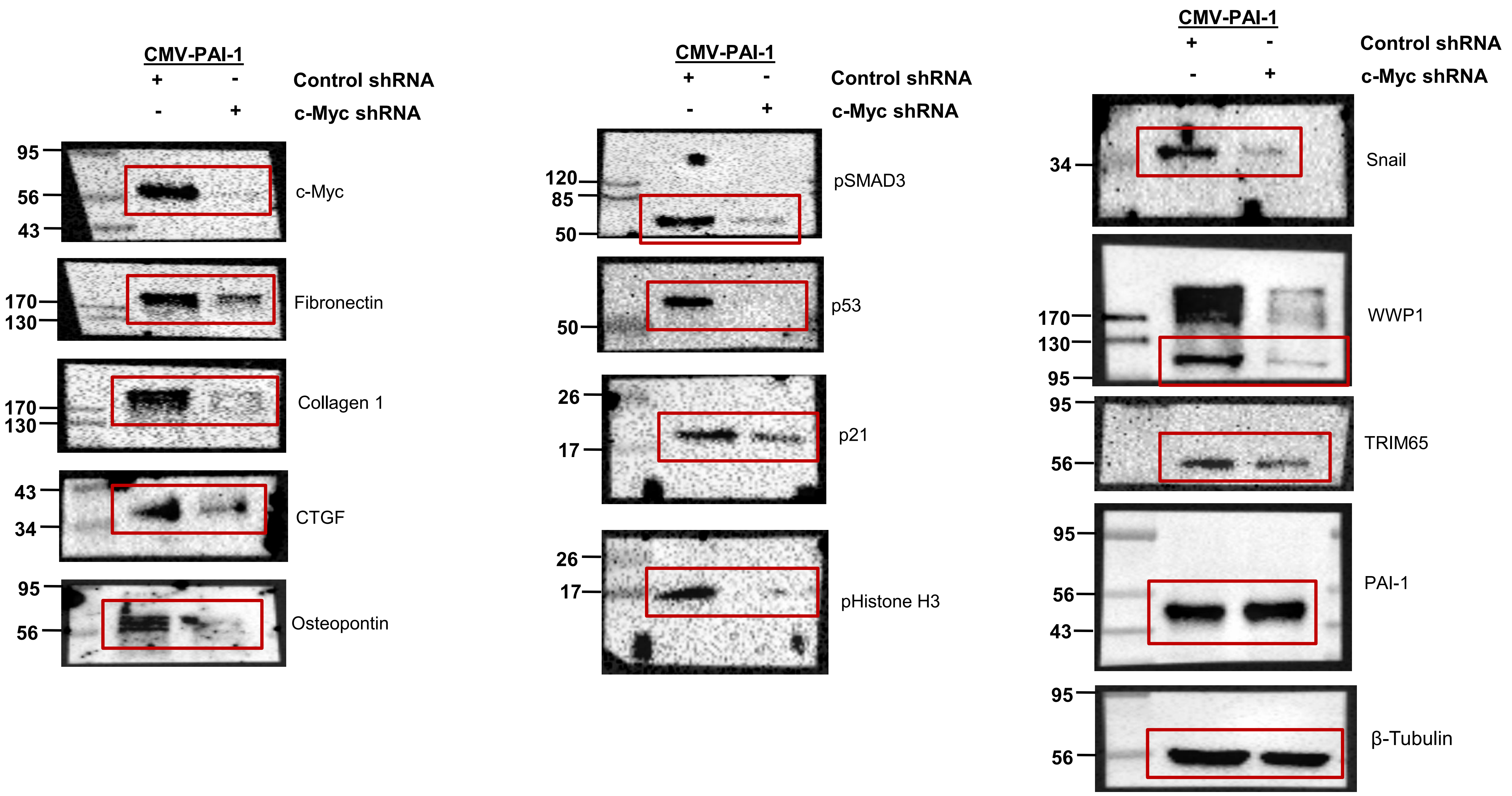

Supplementary Figure S3: Unprocessed original images of blots shown in Figure 7E

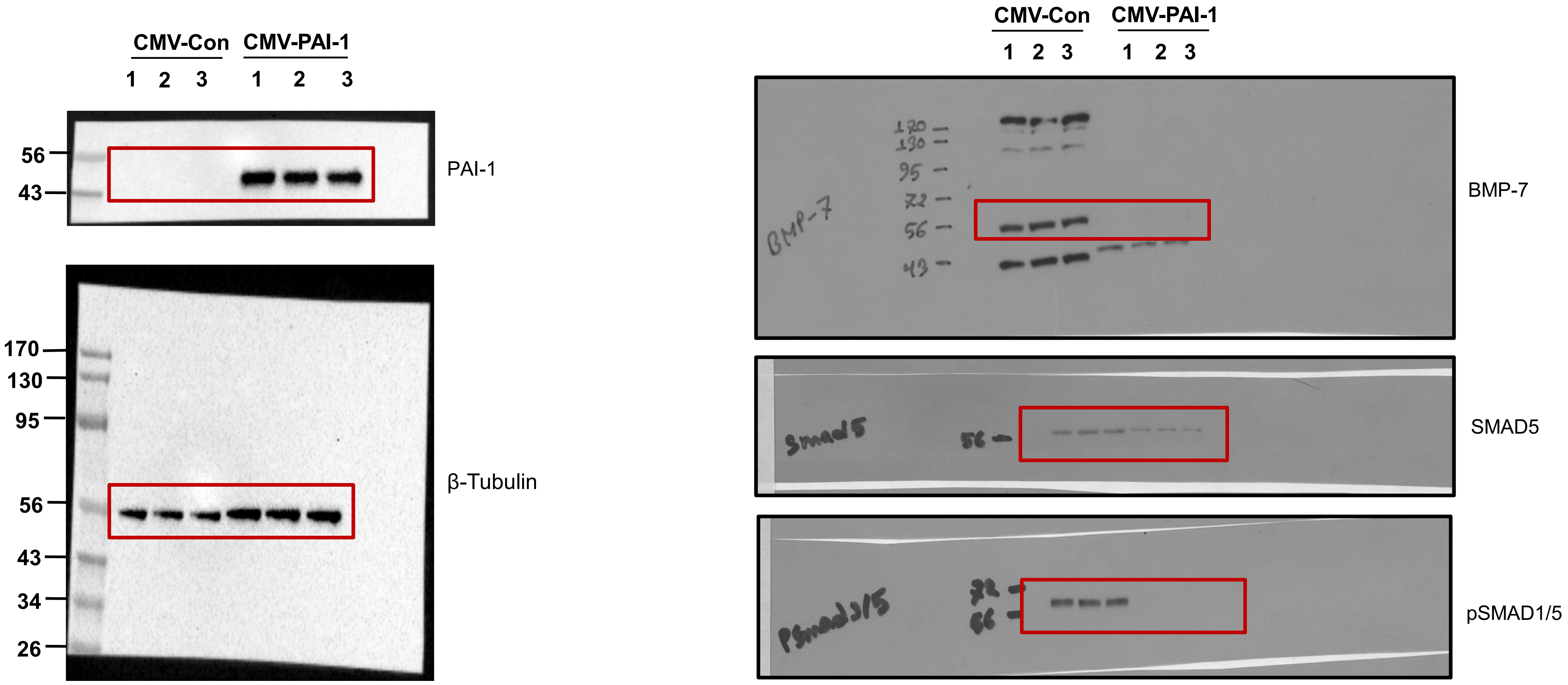

Supplementary Figure S3: Unprocessed original images of blots shown in Figure 9A

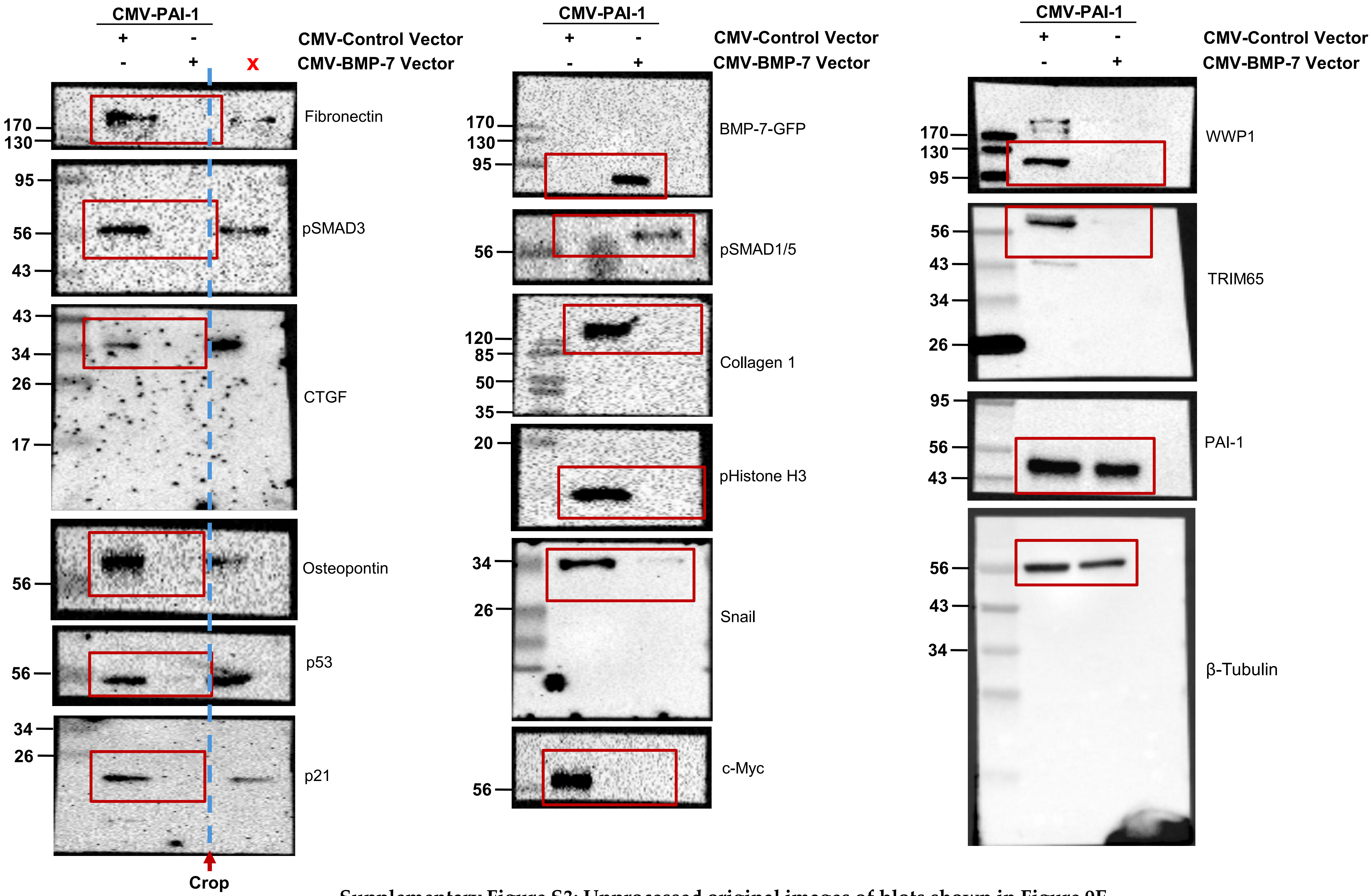

Supplementary Figure S3: Unprocessed original images of blots shown in Figure 9E

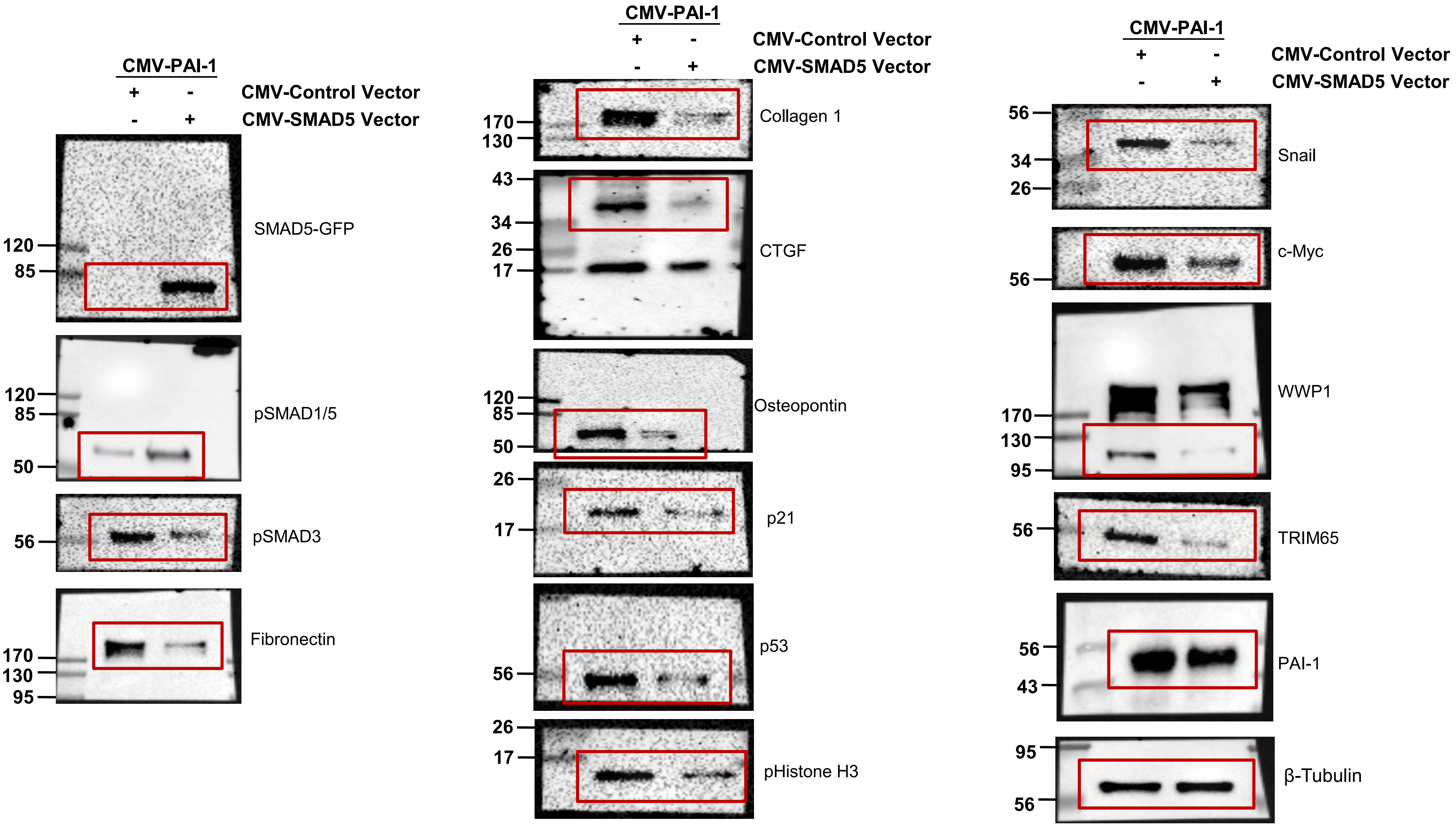

Supplementary Figure S3: Unprocessed original images of blots shown in Figure 10A

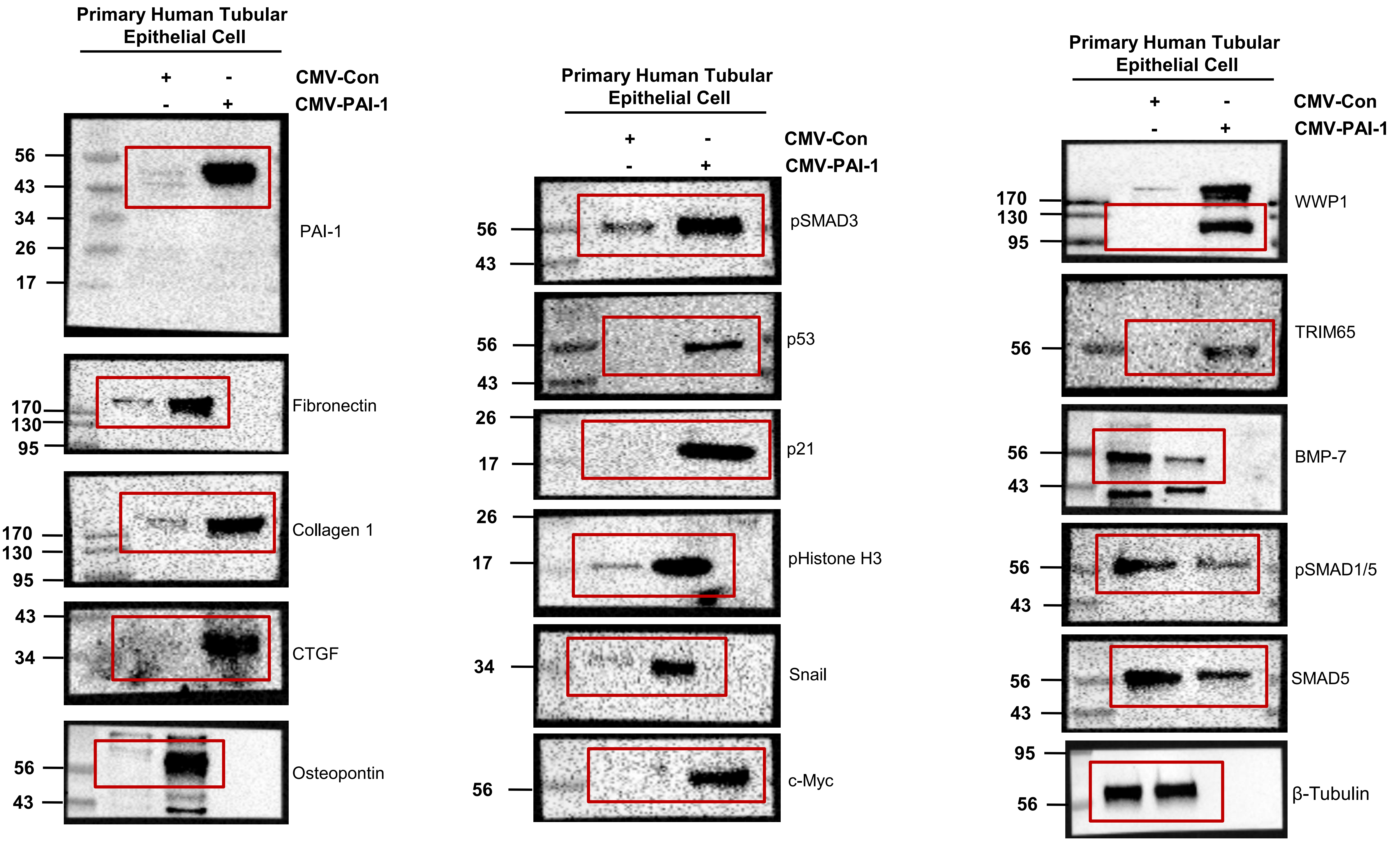

Supplementary Figure S3: Unprocessed original images of blots shown in Supplementary Figure S1

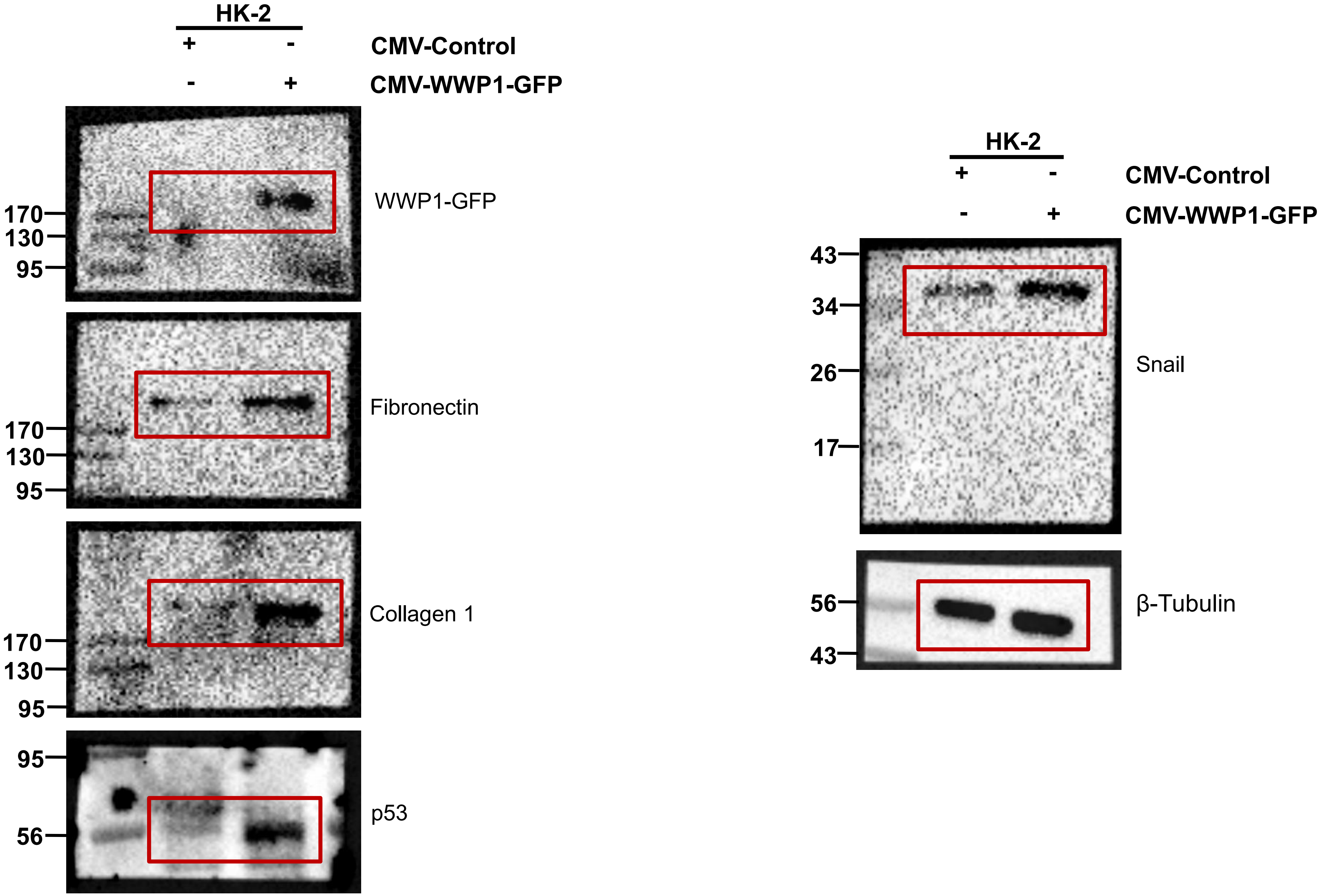

Supplementary Figure S3: Unprocessed original images of blots shown in Supplementary Figure S2
